# Supplementary material for: Machine-Learning-Based Clinical Biomarker Using Cell-Free DNA for Hepatocellular Carcinoma (HCC)
Source: Cancers (Basel). 2022 Apr 20;14(9):2061. doi: 10.3390/cancers14092061 (PMC9103537; doi:10.3390/cancers14092061)
Supplement: Supplementary file 1 [file cancers-14-02061-s001.zip › cancers-1647808-supplementary.pdf]

cancers

Supplementary Material

1 Supplementary Figures and Tables

1.1 Supplementary Figures

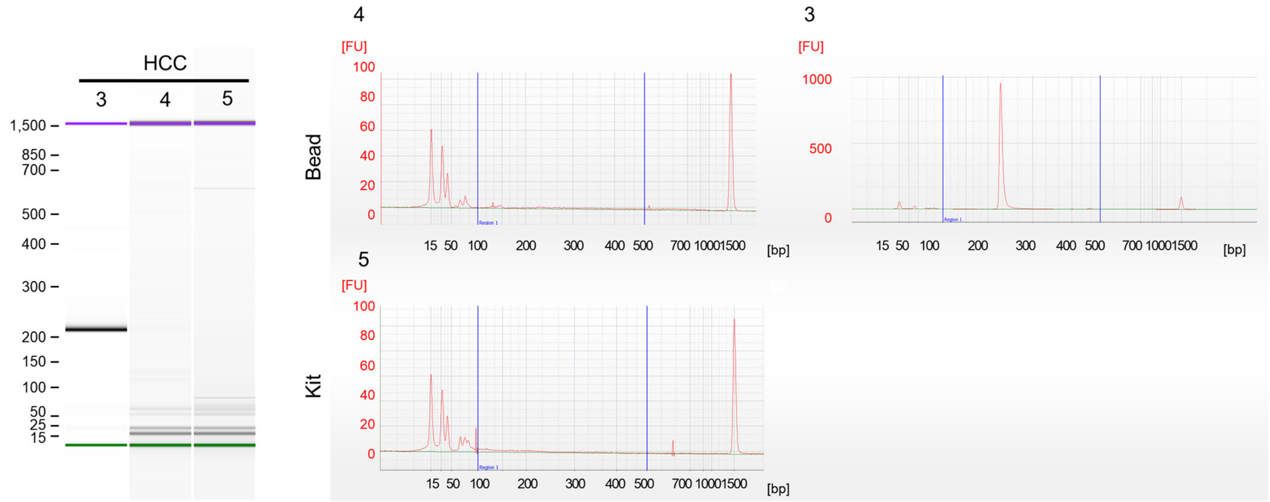

Supplementary Figure S1. Representative electropherograms and gel-like images of cfDNA obtained from HCC.

1.2

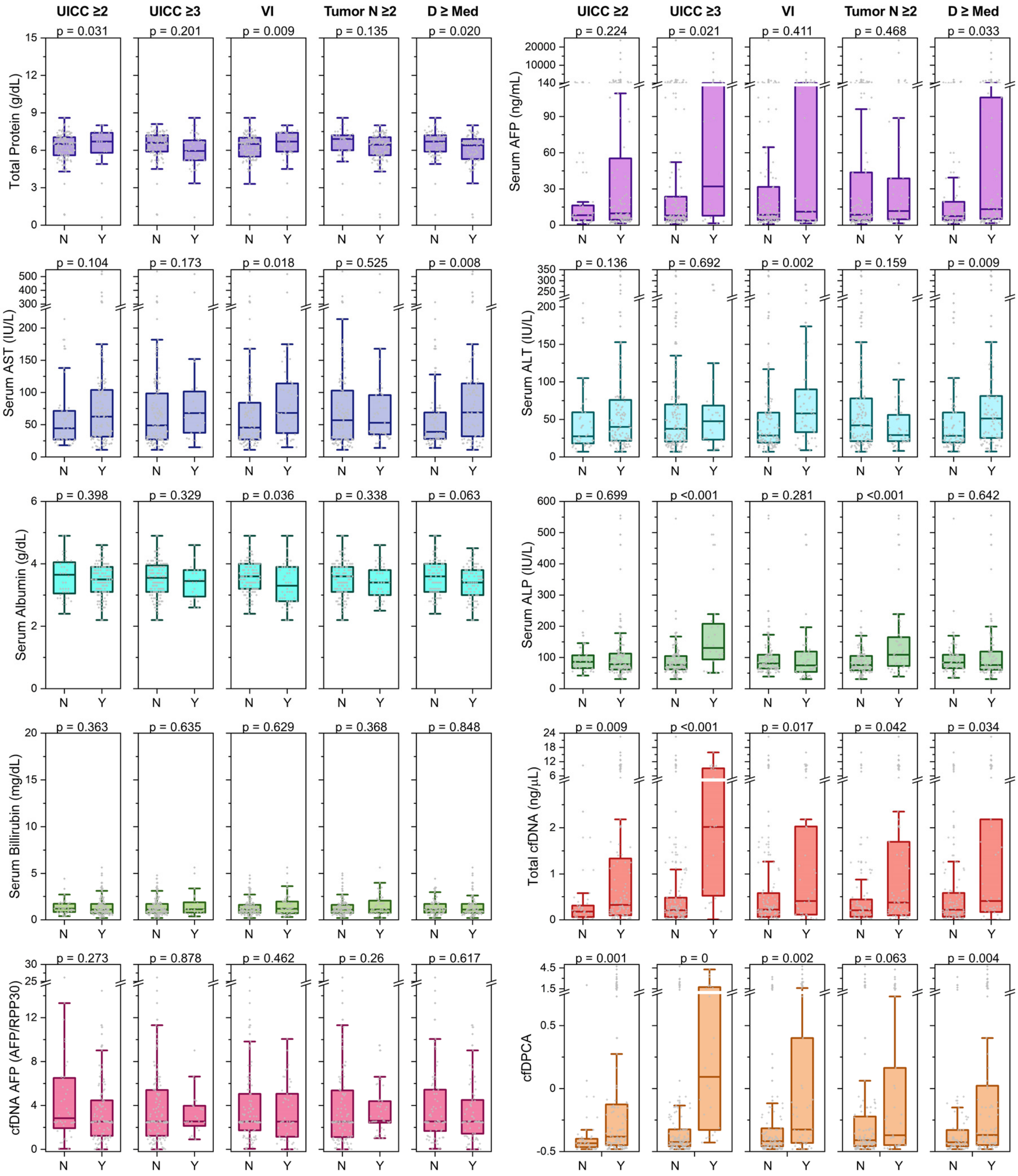

Supplementary Figure S2. The expression profiles of each biomarker depending on patients' pathological features.

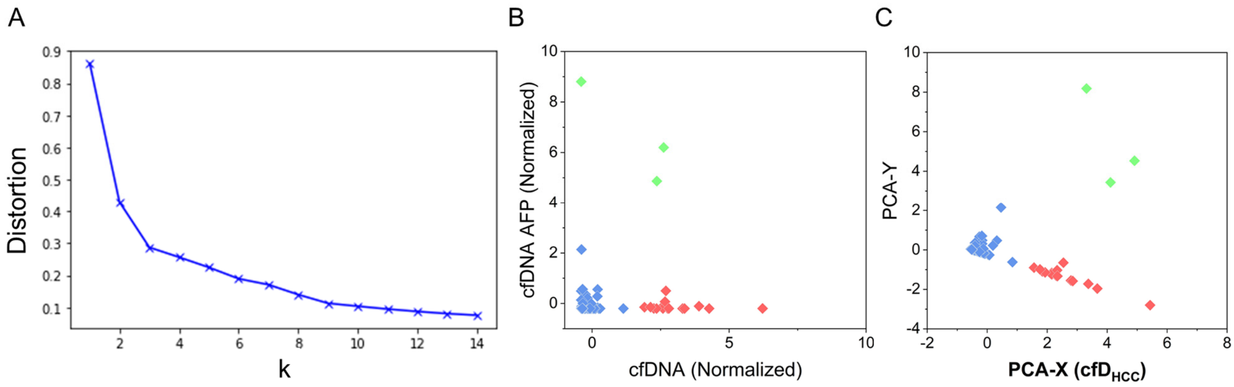

Supplementary Figure S3. Elbow plot for determining the optimal *k* for *k*-means clustering. (A) Elbow plot for determining the optimal *k* for *k*-means clustering of HCC patients based on the expression profiles of cfDNA and relative amount of cfAFP DNA. (B) The expression profiles of cfDNA and cfAFP DNA for each cluster. (C) PCA applied to provide the best linear approximation for stratifying the clusters for cancer patients. The x-axis of the plot (PCA-X) was defined as cfD_{HCC} Score, which was found to minimize the mean-squared reconstruction error of the clusters. Note that blue, red, and green represent cluster group 1, 2, and 3, respectively.

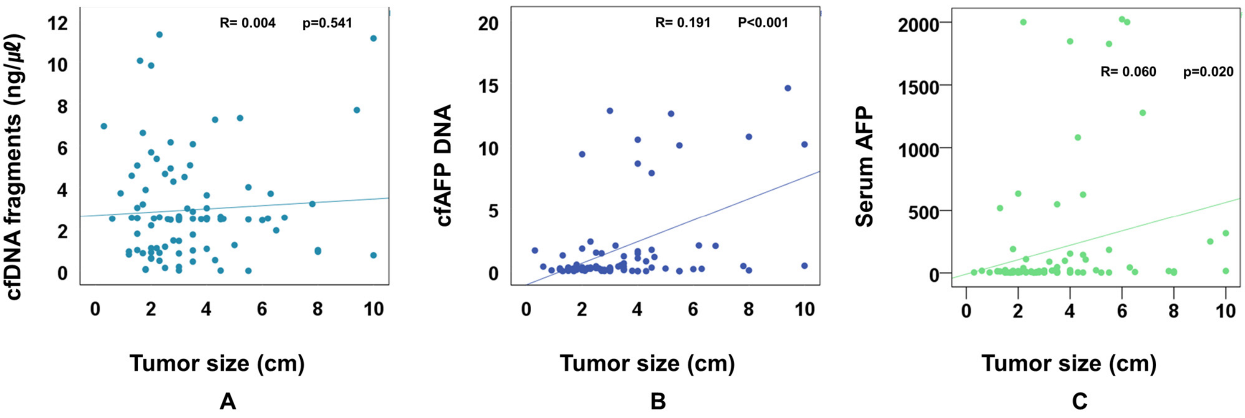

Supplementary Figure S4. Tumor size showed diverse positive correlation coefficient. The amount of plasma cfDNA fragments (A). cfAFP DNA (B). Serum AFP (C). The cfAFP DNA on PDA/silica-coated alginate beads showed a positive correlation coefficient with the tumor size, with Pearson's correlation coefficient of < 0.001.

1.3 Supplementary Tables

Supplementary Table S1. Demographic characteristics of the recruited cancer patients and non-cancer cohorts.

Cohorts	Sex	Age (Years), Median (Range)	Height (cm), Median (Range)	Weight (kg), Median (Range)	Hyper-tension	Diabetes	Alcohol	Smoke	HBV Infection
Cancer	Female: 32 Male: 120 (Total: 152)	62 (46-85)	165 (144-186)	65 (41-120)	61/152 (40.1%)	53/152 (34.9%)	47/152 (30.9%)	53/152 (34.9%)	88/152 (57.9%)
Liver Cirrhosis	Female: 10 Male: 33 (Total: 43)	59 (31-75)	164 (144-177)	61 (41-104)	8/43 (18.6%)	11/43 (25.6%)	21/43 (48.8%)	9/43 (20.9%)	7/43 (16.3%)
Alcoholic Liver Hepatitis	Female: 5 Male: 19 (Total: 24)	51.5 (28-76)	167.6 (146-175)	67 (50-110.8)	2/24 (8.3%)	4/24 (16.7%)	19/24 (79.2%)	12/24 (50%)	N/A
Healthy Donors	Female: 29 Male: 1 (Total: 30)	52.5 (33-70)	155.5 (144-172)	59.5 (46-78)	9/30 (30%)	5/30 (16.7%)	10/30 (33.3%)	0/30 (0%)	0/30 (0%)

Supplementary Table S2. Expression profiles of cfDNA and serum enzymes for the recruited cancer patients and non-cancer cohorts.

Cohorts	cfDNA		Proteins (Enzymes)						
	Total DNA (ng/µL)	AFP (vs.RPP30)	Total Protein (g/dL)	AFP (ng/mL)	AST (IU/L)	ALT (IU/L)	Albumin (g/dL)	ALP (IU/L)	Bilirubin (mg/dL)
HCC	0.25 (0-22.5)	2.53 (0-26.7)	6.5 (0.63-8.6)	9.12 (0.91-23683)	55 (11-538)	38.5 (7-344)	3.5 (2.2-4.9)	80 (31-555)	1.1 (0.19-5.64)
Liver Cirrhosis	0.18 (0-15.22)	0.93 (0.01-14.06)	6.9 (4.9-10.1)	4.2 (0.63-130)	39 (19-159)	26 (10-94)	3.3 (1.8-4.9)	282 (130-535)	1.4 (0.24-19.55)
Alcoholic Liver Hepatitis	0.11 (0-0.29)	0.41 (0-3.95)	6.3 (4.9-8.1)	2.7 (1.2-155)	58.5 (24-360)	26.5 (13-115)	2.65 (1.8-4.5)	107.5 (63-214)	2.3 (1.2-18.0)
Healthy Donors	0.06 (0-0.3)	0.04 (0.01-0.05)	7.3 (6.3-8.2)	2.95 (1.2-8)	7.3 (6.3-8.2)	19 (7-34)	4.6 (4.0-5.1)	72.50 (46-134)	0.76 (0.35-1.93)

Supplementary Table S3. Liquid biopsy marker level from cfDNA, cfAFP DNA, and serum AFP for all patients.

	**Age**	**cfDNA Concentration**	**cfDNA(AFP)**	**serum AFP**
HCC1	56.00	0.48	5.06	20.40
HCC2	64.00	0.00	4.56	11.63
HCC3	78.00	12.57	7.32	2.42
HCC4	69.00	0.24	1.43	5.44
HCC5	46.00	0.29	5.05	9.13
HCC6	66.00	0.25	2.52	15.85
HCC7	59.00	0.37	2.99	18.34
HCC8	60.00	2.03	4.50	88.76
HCC9	54.00	0.00	7.24	4.60
HCC10	60.00	0.17	2.17	3.12
HCC11	49.00	0.21	1.23	21.11
HCC12	46.00	0.39	4.65	9.80
HCC13	67.00	14.61	7.70	249.51
HCC14	56.00	0.27	5.68	634.00
HCC15	59.00	0.01	4.28	4.40
HCC16	49.00	0.16	10.06	3.80
HCC17	52.00	0.43	0.74	315.56
HCC18	55.00	0.22	6.61	1.91
HCC19	46.00	0.29	0.83	3.60
HCC20	60.00	1.42	4.91	5.68
HCC21	50.00	1.64	6.93	4.00
HCC22	50.00	0.00	0.00	1,826.73

HCC23	70.00	0.00	0.02	17.28
HCC24	47.00	0.11	1.45	14.12
HCC25	58.00	0.19	5.37	2,000.00
HCC26	48.00	0.31	0.58	7.96
HCC27	51.00	0.19	0.99	4.00
HCC28	68.00	10.05	4.01	183.39
HCC29	68.00	0.18	1.77	22.45
HCC30	52.00	22.50	1.93	7.54
HCC31	59.00	10.74	0.91	12.28
HCC32	53.00	0.29	0.48	109.19
HCC33	74.00	0.21	0.88	10.67
HCC34	48.00	0.06	3.72	4.08
HCC35	58.00	0.11	0.78	11.87
HCC36	53.00	1.40	1.05	11.75
HCC37	63.00	0.11	3.88	3.02
HCC38	66.00	0.06	0.99	1.78
HCC39	84.00	0.10	0.97	2.95
HCC40	63.00	0.08	0.61	14.66
HCC41	53.00	0.09	0.44	4.68
HCC42	60.00	0.62	6.07	46.12
HCC43	80.00	1.27	0.48	2.87
HCC44	48.00	0.25	0.86	8.49
HCC45	66.00	1.47	1.15	6.92
HCC46	72.00	0.24	0.14	11.70
HCC47	55.00	0.18	0.19	5.97
HCC48	64.00	0.52	0.08	189.20
HCC49	72.00	0.30	0.05	16.82
HCC50	74.00	0.38	4.42	64.58

HCC51	59.00	0.08	6.61	6.85
HCC52	61.00	0.00	4.11	23,683.00
HCC53	48.00	10.21	6.41	16,832.00
HCC54	83.00	0.46	9.02	6.20
HCC55	58.00	2.18	1.95	1.49
HCC56	70.00	9.39	6.63	13,333.00
HCC57	57.00	10.19	3.01	17.70
HCC58	55.00	0.00	0.60	43.68
HCC59	58.00	8.99	1.23	9.50
HCC60	62.00	0.10	1.02	24.38
HCC61	78.00	0.53	5.46	1,478.04
HCC62	54.00	0.28	1.68	59.83
HCC63	69.00	0.35	0.11	1.60
HCC64	63.00	0.26	5.05	4.05
HCC65	67.00	0.41	5.98	4.84
HCC66	63.00	0.44	6.41	0.91
HCC67	69.00	0.70	14.45	3.20
HCC68	57.00	0.64	10.51	40.34
HCC69	66.00	1.25	9.36	3.61
HCC70	82.00	0.39	26.17	4.80
HCC71	70.00	0.14	4.39	8.20
HCC72	70.00	0.00	9.48	6.11
HCC73	60.00	0.32	1.12	6.12
HCC74	84.00	0.00	1.67	5.83
HCC75	66.00	0.78	0.51	1,081.10
HCC76	65.00	0.10	0.84	2.33
HCC77	69.00	0.06	2.43	229.50
HCC78	51.00	0.08	5.92	30.89

HCC79	72.00	0.36	1.73	13.10
HCC80	71.00	0.14	1.80	39.31
HCC81	71.00	0.58	13.33	142.86
HCC82	80.00	0.13	11.82	52.19
HCC83	77.00	1.79	9.83	2.08
HCC84	59.00	0.05	3.45	31.70
HCC85	74.00	0.19	11.31	3.40
HCC86	78.00	0.88	6.38	7.36
HCC87	69.00	10.13	11.13	15.79
HCC88	70.00	0.22	3.36	158.32
HCC89	64.00	0.45	7.66	9.10
HCC90	59.00	0.03	6.16	2.95
HCC91	65.00	0.00	7.64	11.51
HCC92	52.00	0.58	3.94	25.50
HCC93	75.00	1.18	2.84	1.74
HCC94	83.00	0.00	5.83	4.86
HCC95	70.00	0.07	5.44	8.80
HCC96	54.00	0.14	6.60	6.70
HCC97	72.00	10.41	7.98	716.33
HCC98	48.00	0.17	9.16	2.96
HCC99	71.00	0.28	0.01	625.79
HCC100	50.00	0.10	2.61	6.37
HCC101	73.00	0.15	2.50	7.60
HCC102	63.00	12.81	2.54	3.71
HCC103	51.00	2.04	2.51	2,000.00
HCC104	60.00	2.35	2.56	5.11
HCC105	57.00	2.01	2.55	1,278.06
HCC106	49.00	0.24	2.61	2.98

HCC107	60.00	10.50	2.54	1,846.58
HCC108	76.00	0.00	2.52	6.20
HCC109	73.00	0.01	1.20	6.84
HCC110	64.00	0.00	3.66	896.03
HCC111	72.00	0.01	2.33	6,160.00
HCC112	61.00	9.35	1.08	7.20
HCC113	64.00	10.88	1.25	9.74
HCC114	75.00	0.06	1.22	2.74
HCC115	51.00	0.25	1.08	38.76
HCC116	60.00	0.22	0.92	14.53
HCC117	61.00	0.03	2.48	96.16
HCC118	50.00	0.18	3.70	43.41
HCC119	68.00	7.84	2.59	143.42
HCC120	52.00	0.03	3.02	2.28
HCC121	71.00	5.22	2.53	4.86
HCC122	57.00	8.60	3.62	153.10
HCC123	62.00	0.03	2.51	6.46
HCC124	64.00	0.00	3.19	4.31
HCC125	68.00	1.10	2.54	2.94
HCC126	64.00	0.49	3.11	4.08
HCC127	85.00	0.00	2.53	2.85
HCC128	62.00	0.34	3.85	5.58
HCC129	48.00	0.66	2.51	85.79
HCC130	72.00	0.02	2.56	3.00
HCC131	73.00	0.36	2.49	17.18
HCC132	48.00	0.40	3.21	14.80
HCC133	62.00	0.00	2.46	3.29
HCC134	52.00	1.02	3.01	3.65

HCC135	64.00	0.01	2.41	8.38
HCC136	73.00	0.12	2.44	22.90
HCC137	48.00	0.04	2.53	14.61
HCC138	74.00	0.00	2.54	6.62
HCC139	72.00	1.70	2.46	3.07
HCC140	47.00	0.03	2.43	19.24
HCC141	60.00	1.58	2.46	22.19
HCC142	66.00	0.00	2.43	8.66
HCC143	62.00	15.88	2.47	3.59
HCC144	62.00	0.22	2.48	1.82
HCC145	53.00	0.03	2.53	4.12
HCC146	71.00	1.12	2.49	105.70
HCC147	55.00	1.25	2.54	518.50
HCC148	74.00	0.18	2.49	549.20
HCC149	62.00	0.16	2.42	2,023.00
HCC150	48.00	1.09	2.48	10.23
HCC151	58.00	0.21	2.49	7.18
HCC152	57.00	0.12	2.45	5.72
LC1	48.00	0.00	1.08	8.20
LC2	50.00	0.52	0.93	13.00
LC3	50.00	0.00	0.02	1.50
LC4	68.00	1.14	0.01	5.20
LC5	65.00	0.30	0.03	8.10
LC6	59.00	0.65	0.02	5.30
LC7	53.00	0.40	0.02	3.40
LC8	47.00	0.30	0.01	4.20
LC9	70.00	0.02	0.03	2.90
LC10	51.00	0.81	0.02	1.20

LC11	68.00	0.33	0.01	5.70
LC12	56.00	0.50	0.40	6.00
LC13	72.00	0.00	0.05	57.20
LC14	63.00	0.00	0.05	5.20
LC15	75.00	8.80	0.07	3.80
LC16	68.00	0.00	0.97	2.90
LC17	53.00	0.44	0.82	4.80
LC18	63.00	0.08	0.15	2.82
LC19	59.00	0.27	0.92	0.63
LC20	61.00	0.98	0.91	1.80
LC21	62.00	10.52	0.73	8.90
LC22	42.00	0.13	6.82	3.50
LC23	31.00	0.11	0.96	42.70
LC24	46.00	0.00	0.88	12.60
LC25	69.00	0.04	2.13	2.50
LC26	57.00	0.00	8.30	4.40
LC27	50.00	0.44	0.99	3.20
LC28	52.00	0.06	2.22	7.20
LC29	44.00	0.00	1.67	2.60
LC30	75.00	11.49	1.89	130.00
LC31	61.00	0.00	14.06	2.50
LC32	42.00	0.00	2.99	3.90
LC33	42.00	0.00	3.24	2.60
LC34	71.00	0.30	1.11	9.70
LC35	75.00	0.19	1.22	3.10
LC36	73.00	0.06	0.92	3.70
LC37	46.00	0.07	6.67	3.70
LC38	60.00	0.00	3.37	24.80

LC39	38.00	0.20	2.68	3.46
LC40	62.00	11.05	0.87	8.89
LC41	52.00	0.18	1.06	6.43
LC42	57.00	15.22	1.39	1.98
LC43	60.00	0.00	8.79	11.54
LA1	55.00	0.12	1.14	5.30
LA2	50.00	0.00	1.11	2.80
LA3	28.00	0.00	1.64	1.30
LA4	38.00	0.00	1.26	2.20
LA5	43.00	0.11	1.24	155.00
LA6	44.00	0.10	3.12	2.30
LA7	41.00	0.16	3.95	3.00
LA8	76.00	0.04	0.97	4.40
LA9	58.00	0.25	0.81	1.30
LA10	56.00	0.17	0.90	9.50
LA11	44.00	0.04	1.01	3.00
LA12	51.00	0.06	1.54	5.30
LA13	52.00	0.16	0.00	2.50
LA14	75.00	0.16	0.00	1.90
LA15	56.00	0.25	0.01	1.60
LA16	64.00	0.29	0.00	4.30
LA17	48.00	0.03	0.01	3.20
LA18	58.00	0.02	0.01	7.40
LA19	40.00	0.11	0.00	1.20
LA20	72.00	0.18	0.00	2.70
LA21	55.00	0.16	0.00	1.20
LA22	62.00	0.23	0.00	2.70
LA23	47.00	0.00	0.00	2.50

LA24	48.00	0.22	0.00	2.10
HC1	55.00	0.21	0.03	3.50
HC2	38.00	0.00	0.02	1.20
HC3	53.00	0.10	0.01	4.60
HC4	55.00	0.06	0.03	2.10
HC5	55.00	0.10	0.03	4.20
HC6	50.00	0.30	0.01	2.60
HC7	56.00	0.00	0.01	4.60
HC8	49.00	0.07	0.03	7.10
HC9	52.00	0.06	0.01	3.30
HC10	55.00	0.00	0.02	6.10
HC11	54.00	0.20	0.04	2.80
HC12	59.00	0.02	0.02	4.80
HC13	52.00	0.09	0.02	4.40
HC14	50.00	0.08	0.02	7.20
HC15	61.00	0.11	0.13	1.60
HC16	48.00	0.07	0.39	3.60
HC17	60.00	0.18	0.78	2.40
HC18	45.00	0.07	0.10	2.20
HC19	35.00	0.00	0.10	3.10
HC20	66.00	0.00	0.03	2.20
HC21	33.00	0.03	0.05	1.20
HC22	51.00	0.08	0.11	1.50
HC23	63.00	0.00	0.14	1.60
HC24	35.00	0.00	0.27	7.50
HC25	67.00	0.00	0.14	6.60
HC26	49.00	0.00	0.28	2.40
HC27	65.00	0.00	0.12	1.60

HC28	70.00	0.03	0.06	1.80
HC29	42.00	0.06	0.07	1.60
HC30	50.00	0.00	0.05	3.80

Supplementary Table S4. AUC-ROC values for detecting HCC from NC, LC, LA, and HD.

	VS. NC	VS. LC	VS. LA	VS. HD
cfDNA (ng/μL)	0.688 ± 0.034 (0.621-0.755; p <0.0001)	0.713 ± 0.041 (0.632-0.794; p = 0.001)	0.592 ± 0.052 (0.490-0.694; p = 0.066)	0.805 ± 0.034 (0.739-0.871; p <0.0001)
cfAFP-DNA (vs. RPP30)	0.843 ± 0.027 (0.790-0.897; p <0.0001)	0.861 ± 0.040 (0.783-0.940 p <0.0001)	0.744 ± 0.047 (0.651-0.837; p <0.0001)	0.971 ± 0.012 (0.948-0.994; p <0.0001)
Total Protein (g/dL)	0.361± 0.035 (0.292-0.429; p <0.0001)	0.522 ± 0.058 (0.409-0.635; p = 0.727)	0.377 ± 0.0435 (0.288-0.465; p = 0.014)	0.208 ± 0.036 (0.137-0.279; p <0.0001)
Serum AFP (ng/mL)	0.780 ± 0.029 (0.723-0.837; p <0.0001)	0.836± 0.044 (0.750-0.922; p <0.0001)	0.706 ± 0.042 (0.624-0.787; p <0.0001)	0.840 ± 0.032 (0.777-0.904; p <0.0001)
Serum AST (IU/L)	0.638 ± 0.035 (0.569-0.708; p <0.0001)	0.422 ± 0.052 (0.321-0.523; p = 0.221)	0.567 ± 0.043 (0.483-0.651; p = 0.179)	0.913 ± 0.021 (0.872-0.954; p <0.0001)
Serum ALT (IU/L)	0.649 ± 0.035 (0.582-0.718; p <0.0001)	0.582 ± 0.057 (0.470-0.695; p = 0.195)	0.608 ± 0.044 (0.521-0.695; p = 0.031)	0.763 ± 0.036 (0.692-0.833; p = 0.031)
Serum Albumin (g/dL)	0.445 ± 0.042 (0.362-0.528; p = 0.145)	0.813 ± 0.053 (0.708-0.917; p <0.0001)	0.528 ± 0.055 (0.419-0.636; p = 0.582)	0.033 ± 0.012 (0.009-0.057; p <0.0001)
Serum ALP (IU/L)	0.280 ± 0.034 (0.214-0.346; p <0.0001)	0.327 ± 0.048 (0.232-0.422; p = 0.007)	0.044 ± 0.015 (0.016-0.073; p <0.0001)	0.581 ± 0.047 (0.489-0.673; p = 0.161)
Serum Bilirubin (mg/dL)	0.446 ± 0.038 (0.371-0.522; p = 0.152)	0.157 ± 0.036 (0.0.086-0.229; p <0.0001)	0.425 ± 0.054 (0.319-0.531; p = 0.134)	0.707 ± 0.040 (0.629-0.785; p <0.0001)

* NC: Non-cancer cohorts (LC + LA + HD); LC: Liver cirrhosis; LA: Alcoholic liver hepatitis; HD: Healthy donors

Supplementary Table S5. Diagnostic performance of serum enzymes, plasma cfDNA, and cfAFP DNA for detecting HCC patients. Note that the diagnostic performance of each biomarker was determined at the threshold which results in the highest accuracy with specificity higher than 0.7.

	vs. Non-cancer Cohorts (NC)					*vs. Patients with Alcoholic Liver Hepatitis (LA)*				
	Sensitivity	Specificity	Accuracy	PPV	NPV	Sensitivity	Specificity	Accuracy	PPV	NPV
cfDNA	0.579	0.732	0.639	0.772	0.526	0.625	0.792	0.648	0.950	0.250
cfAFP-DNA	0.796	0.784	0.791	0.852	0.710	0.796	0.750	0.790	0.953	0.367
Total Protein	0.007	0.990	0.390	0.500	0.389	0.382	0.750	0.432	0.906	0.161
Serum AFP	0.691	0.753	0.715	0.814	0.608	0.842	0.708	0.824	0.948	0.415
Serum AST	0.493	0.753	0.594	0.758	0.487	0.289	0.750	0.352	0.88	0.143
Serum ALT	0.513	0.763	0.610	0.772	0.500	0.500	0.708	0.528	0.916	0.183
Serum Albumin	0	0.990	0.386	0	0.387	0.809	0.708	0.795	0.946	0.37
Serum ALP	0.013	1	0.398	1	0.393	0.224	0.708	0.290	0.829	0.126
Serum Bilirubin	0	0.990	0.386	0	0.387	0	0.958	0.131	0	0.131

	vs. Patients with Liver Cirrhosis (LC)					*vs. Healthy Donors (HD)*				
	Sensitivity	Specificity	Accuracy	PPV	NPV	Sensitivity	Specificity	Accuracy	PPV	NPV
cfDNA	0.355	0.744	0.441	0.831	0.246	0.750	0.767	0.753	0.942	0.377
cfAFP-DNA	0.743	0.721	0.738	0.904	0.443	0.947	0.967	0.951	0.993	0.784
Total Protein	0.164	0.791	0.303	0.735	0.211	0.033	0.967	0.187	0.833	0.165
Serum AFP	0.566	0.721	0.600	0.878	0.320	0.730	0.800	0.742	0.949	0.369
Serum AST	0.474	0.744	0.533	0.867	0.286	0.855	0.867	0.857	0.970	0.542
Serum ALT	0.421	0.814	0.508	0.889	0.285	0.658	0.767	0.676	0.935	0.307
Serum Albumin	0.151	0.744	0.282	0.676	0.199	0	0.967	0.159	0	0.16
Serum ALP	0.013	1	0.231	1	0.223	0.434	0.733	0.484	0.892	0.204
Serum Bilirubin	0.158	0.721	0.282	0.667	0.195	0.618	0.833	0.654	0.949	0.301

Supplementary Table S6. Clinical characteristics of recruited HCC patients. Note that the numbers represent the median (IQR) for each biomarker used in this study.

	No. of Patients	cfD_{HCC} Score (AU)	cfDNA (ng/μL)	cfAFP DNA (vs. RPP30)	Total Protein (g/dL)	AFP (ng/mL)	AST (IU/L)	ALT (IU/L)	Albumin (g/dL)	ALP (IU/L)	Bilirubin (mg/dL)
UICC Stage											
I	40	-0.40 ± 0.46	0.55 ± 1.67	4.68 ± 4.81	6.51 ± 1.35	56.82 ± 151.46	60.77 ± 50.35	48.26 ± 48.01	3.57 ± 0.63	93.65 ± 42.26	1.37 ± 0.81
II	88	-0.06 ± 1.1	1.57 ± 3.91	3.39 ± 2.92	6.12 ± 1.05	436.91 ± 2546.82	81.04 ± 84.71	64.46 ± 68.99	3.48 ± 0.52	86.88 ± 60.65	1.32 ± 0.93
III	17	0.26 ± 1.09	2.65 ± 4.17	2.77 ± 1.04	6.69 ± 0.90	623.52 ± 1579.16	85.44 ± 87.14	62.44 ± 65.38	3.51 ± 0.51	136.31 ± 87.42	1.42 ± 1.08
IV	7	2.14 ± 1.80	7.78 ± 4.05	4.15 ± 2.93	5.60 ± 2.47	3799.45 ± 7026.81	132.38 ± 161.37	67.63 ± 82.63	3.19 ± 0.51	277.00 ± 194.10	1.97 ± 1.83
TACE Treated											
No	109	0.01 ± 1.13	1.87 ± 3.93	3.36 ± 3.38	6.20 ± 1.13	352.60 ± 1738.42	88.63 ± 94.21	72.41 ± 71.21	3.50 ± 0.57	90.95 ± 69.67	1.34 ± 0.96
Yes	43	-0.01 ± 1.21	1.42 ± 3.44	4.55 ± 3.48	6.39 ± 1.49	992.07 ± 4095.92	54.14 ± 38.18	29.07 ± 20.81	3.46 ± 0.51	136.60 ± 105.31	1.49 ± 1.05
LVI											
No	106	-0.20 ± 0.82	1.13 ± 2.78	3.86 ± 3.73	6.40 ± 1.15	333.7 ± 2315.74	72.30 ± 80.13	53.48 ± 63.69	3.56 ± 0.50	104.08 ± 79.70	1.32 ± 0.87
Yes	46	0.45 ± 1.60	3.16 ± 5.23	3.32 ± 2.62	5.92 ± 1.38	993.92 ± 3219.91	94.01 ± 90.34	75.51 ± 63.63	3.34 ± 0.63	103.39 ± 92.78	1.53 ± 1.2
Multifocality											
No	111	-0.11 ± 1.03	1.44 ± 3.63	3.79 ± 3.82	6.21 ± 1.20	329.05 ± 1431.91	78.07 ± 80.12	65.80 ± 69.12	3.52 ± 0.56	84.34 ± 37.91	1.31 ± 0.91
Yes	41	0.31 ± 1.39	2.56 ± 4.14	3.47 ± 2.07	6.39 ± 1.35	1087.01 ± 4474.57	81.05 ± 93.55	44.85 ± 46.13	3.42 ± 0.52	156.73 ± 135.95	1.56 ± 1.14
Tumor Size											
< 2.3 cm	73	-0.26 ± 0.75	1.05 ± 2.80	4.06 ± 4.10	6.48 ± 1.14	84.61 ± 279.16	59.87 ± 53.06	47.58 ± 49.56	3.59 ± 0.56	96.70 ± 65.41	1.32 ± 0.8
≥ 2.3 cm	79	0.24 ± 1.38	2.39 ± 4.44	3.37 ± 2.66	6.05 ± 1.31	948.31 ± 3597.9	96.43 ± 101.47	71.76 ± 73.79	3.40 ± 0.53	110.49 ± 97.34	1.43 ± 1.12

Supplementary Table S7. AUC-ROC values of the biomarkers for determining the pathological features of HCC tumors.

	UICC ≥2	UICC ≥3	VI	TUMOR N ≥2	SIZE ≥MEDIAN
cfDHCC (Normalized)	0.673 ± 0.046 (0.583-0.763; p = 0.001)	0.835 ± 0.038 (0.762-0.909; p <0.001)	0.656 ± 0.051 (0.557-0.756; p = 0.002)	0.598 ± 0.053 (0.494-0.703; p = 0.063)	0.635 ± 0.045 (0.546-0.723; p = 0.004)
cfDNA (ng/µL)	0.639 ± 0.047 (0.546-0.731; p = 0.009)	0.819 ± 0.045 (0.730-0.907; p <0.001)	0.622 ± 0.052 (0.520-0.725; p = 0.017)	0.608 ± 0.053 (0.503-0.712; p = 0.042)	0.600 ± 0.046 (0.510-0.690; p = 0.034)
cfAFP DNA (vs. RPP30)	0.441 ± 0.056 (0.332-0.550; p = 0.268)	0.51 ± 0.054 (0.405-0.616; p =0.876)	0.462 ± 0.053 (0.358-0.567; p = 0.461)	0.56 ± 0.047 (0.468-0.651; p = 0.259)	0.476 ± 0.047 (0.384-0.568; p = 0.608)
Total Protein (g/dL)	0.385 ± 0.052 (0.282-0.487; p = 0.031)	0.582 ± 0.069 (0.447-0.718; p =0.201)	0.366 ± 0.052 (0.264-0.468; p = 0.009)	0.579 ± 0.053 (0.474-0.684; p = 0.135)	0.391 ± 0.046 (0.302-0.481; p = 0.021)
Serum AFP (ng/mL)	0.565 ± 0.050 (0.467-0.663; p = 0.224)	0.649 ± 0.066 (0.519-0.779; p =0.021)	0.542 ± 0.055 (0.435-0.649; p = 0.411)	0.538 ± 0.052 (0.437-0.640; p = 0.468)	0.600 ± 0.046 (0.51-0.69; p = 0.033)
Serum AST (IU/L)	0.587 ± 0.052 (0.485-0.688; p = 0.104)	0.588 ± 0.06 (0.471-0.705; p =0.173)	0.621 ± 0.049 (0.525-0.717; p = 0.018)	0.534 ± 0.049 (0.437-0.630; p = 0.525)	0.624 ± 0.046 (0.533-0.714; p = 0.008)
Serum ALT (IU/L)	0.580 ± 0.052 (0.477-0.682; p = 0.136)	0.526 ± 0.065 (0.398-0.653; p =0.692)	0.658 ± 0.049 (0.563-0.753; p = 0.002)	0.425 ± 0.049 (0.330-0.521; p = 0.159)	0.622 ± 0.046 (0.533-0.712; p = 0.009)
Serum Albumin (g/dL)	0.455 ± 0.057 (0.342-0.568; p = 0.399)	0.437 ± 0.061 (0.318-0.556; p =0.329)	0.393 ± 0.0540 (0.288-0.498; p = 0.037)	0.449 ± 0.052 (0.348-0.551; p = 0.339)	0.413 ± 0.046 (0.322-0.503; p = 0.064)
Serum ALP (IU/L)	0.479 ± 0.050 (0.381-0.578; p = 0.699)	0.743 ± 0.067 (0.611-0.875; p <0.001)	0.445 ± 0.055 (0.336-0.553; p = 0.281)	0.696 ± 0.051 (0.596-0.797; p <0.001)	0.478 ± 0.047 (0.385-0.571; p = 0.642)
Serum Bilirubin (mg/dL)	0.451 ± 0.051 (0.352-0.551; p = 0.363)	0.531 ± 0.066 (0.401-0.660; p =0.635)	0.525 ± 0.054 (0.419-0.631; p = 0.629)	0.548 ± 0.055 (0.441-0.655; p = 0.368)	0.491 ± 0.047 (0.399-0.583; p = 0.848)

* UICC: The cancer stage defined by union for international cancer control; VI: Vascular invasion; Tumor N: Number of tumors.

Supplementary Table S8. Diagnostic performance of serum enzymes, plasma cfDNA, and cfAFP DNA for determining the pathological features of HCC patients (Threshold: median expression level of each biomarker).

	UICC ≥2					*UICC ≥3*				
	Sensitivity	Specificity	Accuracy	PPV	NPV	Sensitivity	Specificity	Accuracy	PPV	NPV
cfDHCC	0.580	0.725	0.618	0.855	0.382	0.917	0.578	0.632	0.289	0.974
cfDNA	0.563	0.650	0.586	0.818	0.347	0.917	0.570	0.625	0.286	0.973
cfAFP DNA	0.482	0.450	0.474	0.711	0.237	0.500	0.500	0.500	0.158	0.842
Total Protein	0.500	0.350	0.461	0.683	0.200	0.625	0.477	0.500	0.183	0.871
Serum AFP	0.527	0.575	0.539	0.776	0.303	0.750	0.547	0.579	0.237	0.921
Serum AST	0.527	0.575	0.539	0.776	0.303	0.625	0.523	0.539	0.197	0.882
Serum ALT	0.518	0.550	0.526	0.763	0.289	0.542	0.508	0.513	0.171	0.855
Serum Albumin	0.527	0.475	0.513	0.738	0.264	0.500	0.469	0.474	0.150	0.833
Serum ALP	0.491	0.450	0.480	0.714	0.240	0.792	0.547	0.586	0.247	0.933
Serum Bilirubin	0.500	0.400	0.474	0.700	0.222	0.542	0.477	0.487	0.163	0.847

	VI					*Tumor Number ≥2*				
	Sensitivity	Specificity	Accuracy	PPV	NPV	Sensitivity	Specificity	Accuracy	PPV	NPV
cfDHCC	0.652	0.566	0.592	0.395	0.789	0.634	0.550	0.572	0.342	0.803
cfDNA	0.609	0.538	0.559	0.364	0.760	0.634	0.541	0.566	0.338	0.800
cfAFP DNA	0.478	0.491	0.487	0.289	0.684	0.610	0.541	0.559	0.329	0.789
Total Protein	0.413	0.406	0.408	0.232	0.614	0.561	0.468	0.493	0.280	0.743
Serum AFP	0.565	0.528	0.539	0.342	0.737	0.561	0.523	0.533	0.303	0.763
Serum AST	0.674	0.575	0.605	0.408	0.803	0.488	0.495	0.493	0.263	0.724
Serum ALT	0.696	0.585	0.618	0.421	0.816	0.366	0.450	0.428	0.197	0.658
Serum Albumin	0.435	0.434	0.434	0.250	0.639	0.463	0.450	0.454	0.238	0.694
Serum ALP	0.478	0.481	0.480	0.286	0.680	0.683	0.559	0.592	0.364	0.827
Serum Bilirubin	0.565	0.491	0.513	0.325	0.722	0.537	0.477	0.493	0.275	0.736

	Tumor Diameter ≥ Median				
	Sensitivity	Specificity	Accuracy	PPV	NPV
cfDHCC	0.608	0.616	0.612	0.632	0.592
cfDNA	0.570	0.562	0.566	0.584	0.547
cfAFP DNA	0.481	0.479	0.480	0.500	0.461
Total Protein	0.494	0.411	0.454	0.476	0.429
Serum AFP	0.570	0.575	0.572	0.592	0.553
Serum AST	0.608	0.616	0.612	0.632	0.592
Serum ALT	0.595	0.603	0.599	0.618	0.579
Serum Albumin	0.494	0.438	0.467	0.488	0.444
Serum ALP	0.468	0.452	0.461	0.481	0.440
Serum Bilirubin	0.519	0.466	0.493	0.513	0.472

Supplementary Table S9. Odd ratio of serum enzymes, plasma cfDNA, and cfAFP DNA for determining the pathological features of HCC patients (Threshold: median expression level of each biomarker).

	cfD_{HCC} Score	cfDNA	cfAFP DNA	Total Protein	AFP	AST	ALT	Albumin	ALP	Bilirubin
UICC ≥2	3.646 (1.656-8.026) p = 0.002	2.388 (1.129-5.052) p = 0.027	0.762 (0.369-1.573) p = 0.581	0.538 (0.255-1.138) p = 0.139	1.506 (0.727-3.120) p = 0.357	1.506 (0.727-3.120) p = 0.357	1.313 (0.636-2.710) p = 0.581	1.007 (0.489-2.075) p = 1.000	0.789 (0.382-1.63) p = 0.583	0.667 (0.32-1.388) p = 0.357
UICC ≥3	15.074 (3.399-66.847) p <0.001	14.600 (3.293-64.732) p <0.001	1.000 (0.418-2.392) p = 1.000	1.517 (0.619-3.718) p = 0.383	3.621 (1.349-9.718) p = 0.013	1.831 (0.747-4.486) p = 0.266	1.219 (0.509-2.924) p = 0.824	0.882 (0.369-2.111) p = 0.826	4.586 (1.613-13.038) p = 0.003	1.076 (0.449-2.58) p = 1.000
VI	2.446 (1.193-5.015) p = 0.021	1.810 (0.894-3.661) p = 0.114	0.883 (0.442-1.764) p = 0.860	0.480 (0.238-0.971) p = 0.051	1.456 (0.725-2.922) p = 0.377	2.801 (1.354-5.795) p = 0.008	3.221 (1.541-6.733) p = 0.002	0.590 (0.293-1.185) p = 0.159	0.850 (0.425-1.699) p = 0.725	1.252 (0.624-2.512) p = 0.597
Tumor Number ≥2	2.115 (1.012-4.42) p = 0.067	2.039 (0.976-4.261) p = 0.068	1.838 (0.886-3.815) p = 0.143	1.126 (0.548-2.315) p = 0.855	1.398 (0.68-2.874) p = 0.465	0.935 (0.457-1.915) p = 1.000	0.473 (0.226-0.988) p = 0.067	0.708 (0.345-1.453) p = 0.366	2.725 (1.278-5.81) p = 0.010	1.058 (0.516-2.169) p = 1.000
Tumor D ≥ Median	2.488 (1.295-4.781) p = 0.009	1.696 (0.892-3.222) p = 0.144	0.854 (0.452-1.614) p = 0.746	0.680 (0.358-1.292) p = 0.258	1.793 (0.942-3.412) p = 0.104	2.488 (1.295-4.781) p = 0.009	2.228 (1.164-4.266) p = 0.023	0.761 (0.402-1.442) p = 0.421	0.727 (0.384-1.376) p = 0.336	0.941 (0.497-1.779) p = 0.872

* Odd ratio (95%CI)

Supplementary Table S10. Kaplan-Meier survival analysis for RFS of TACE-treated patients.

Biomarkers	Cut-off								
	Q1			Median			Q3		
	≥ Q1	< Q1	P-Value	≥ Q2	< Q2	P-Value	≥ Q3	< Q3	P-Value
**cfD$_{HCC}$ Score**	21.473 ± 3.833 (13.862-29.084)	19.500 ± 6.223 (7.303-31.697)	0.796	15.905 ± 3.988 (8.089-23.721)	26.489 ± 5.221 (16.256-36.721)	0.061	11.636 ± 4.886 (2.059-21.214)	24.559 ± 4.035 (16.651-32.467)	0.034
**cfDNA**	22.634 ± 3.908 (14.975-30.292)	12.667 ± 3.307 (6.184-19.149)	0.348	16.524 ± 3.970 (8.742-24.305)	25.958 ± 5.237 (15.695-36.222)	0.153	16.273 ± 6.188 (4.144-28.401)	22.843 ± 3.969 (15.065-30.622)	0.201
**cfAFP DNA**	22.965 ± 4.127 (14.876-31.053)	15.700 ± 4.847 (6.201-25.199)	0.239	26.578 ± 5.183 (16.420-36.736)	13.794 ± 3.089 (7.741-19.848)	0.051	22.100 ± 6.294 (9.764-34.436)	20.764 ± 4.002 (12.921-28.608)	0.994
**Total Protein**	18.566 ± 3.191 (12.312-24.820)	31.875 ± 9.636 (12.988-50.762)	0.179	20.217 ± 3.796 (12.777-27.658)	22.859 ± 5.850 (11.392-34.325)	0.482	13.083 ± 2.080 (9.007-17.160)	24.619 ± 4.501 (15.797-33.440)	0.189
**AFP**	14.545 ± 3.808 (7.082-22.009)	20.701 ± 3.919 (14.019-29.382)	0.628	17.190 ± 3.966 (9.417-24.964)	25.103 ± 5.332 (14.654-35.553)	0.150	17.868 ± 3.446 (11.113-24.623)	27.545 ± 6.989 (13.847-41.244)	0.200
**AST**	19.144 ± 3.791 (11.713-26.575)	27.644 ± 6.774 (14.367-40.922)	0.471	20.955 ± 4.981 (11.191-30.718)	20.754 ± 4.292 (12.342-29.167)	0.886	13.955 ± 5.815 (2.558-25.351)	23.476 ± 3.971 (15.693-31.260)	0.092
**ALT**	22.171 ± 3.889 (14.549-20.794)	16.625 ± 6.158 (4.554-28.696)	0.308	21.216 ± 4.866 (11.679-30.753)	21.070 ± 4.617 (12.021-30.119)	0.917	22.145 ± 6.298 (9.802-34.489)	20.422 ± 3.859 (12.879-28.006)	0.715
**Albumin**	22.384 ± 3.655 (15.220-29.548)	15.611 ± 7.613 (0.690-30.531)	0.391	20.704 ± 3.872 (13.115-28.293)	21.363 ± 5.776 (10.042-32.684)	0.813	22.667 ± 5.662 (11.648-33.685)	20.266 ± 4.026 (12.375-28.158)	0.654
**ALP**	21.256 ± 3.817 (13.775-28.737)	18.100 ± 6.661 (5.044-31.156)	0.737	20.557 ± 4.472 (11.791-29.322)	21.012 ± 4.835 (11.535-30.489)	0.898	18.273 ± 6.288 (5.948-30.597)	21.727 ± 3.820 (14.239-29.215)	0.876
**Bilirubin**	17.065 ± 3.618 (9.973-24.156)	33.067 ± 6.402 (20.519-45.615)	0.064	17.273 ± 4.179 (9.083-25.463)	25.090 ± 5.053 (15.185-34.994)	0.276	13.364 ± 4.941 (3.678-23.049)	23.694 ± 3.992 (15.869-31.518)	0.196

* Survival duration ± S.D. (95% CI)

** Units: Months

Supplementary Table S11. Kaplan-Meier survival analysis for marginal recurrence of TACE-treated patients.

Biomarkers	Cut-off								
	Q1			Median			Q3		
	≥ Q1	< Q1	P-Value	≥ Q2	< Q2	P-Value	≥ Q3	< Q3	P-Value
**cfD$_{HCC}$ Score**	33.247 ± 5.141 (23.171-43.323)	36.167 ± 9.884 (16.794-55.539)	0.483	27.379 ± 6.314 (15.003-39.755)	41.146 ± 6.333 (28.733-53.559)	0.061	30.222 ± 10.775 (9.104-51.341)	36.360 ± 5.138 (26.288-46.43)	0.228
**cfDNA**	35.887 ± 5.125 (25.842-45.933)	19.400 ± 4.377 (10.822-27.978)	0.592	27.947 ± 6.198 (15.799-40.096)	41.256 ± 6.389 (28.734-53.777)	0.121	37.222 ± 10.493 (16.656-57.788)	34.128 ± 5.191 (23.954-44.302)	0.866
**cfAFP DNA**	34.957 ± 5.246 (24.675-45.239)	27.238 ± 6.994 (13.529-40.947)	0.907	41.597 ± 6.065 (29.709-53.484)	20.387 ± 4.632 (11.308-29.466)	0.094	43.571 ± 9.832 (24.301-62.842)	30.692 ± 5.314 (20.278-41.107)	0.327
**Total Protein**	31.349 ± 5.049 (21.453-41.244)	51.143 ± 7.375 (36.689-65.597)	0.256	31.282 ± 5.624 (20.259-42.306)	41.480 ± 7.300 (27.172-55.788)	0.406	15.295 ± 2.437 (10.519-20.071)	41.081 ± 5.389 (30.519-51.644)	0.066
**AFP**	17.000 ± 4.308 (8.556-25.444)	35.610 ± 5.498 (24.834-46.386)	0.250	61.164 ± 5.384 (15.612-36.716)	41.823 ± 6.465 (29.151-54.494)	0.091	29.049 ± 4.814 (19.614-38.483)	41.804 ± 8.299 (25.538-58.070)	0.252
**AST**	31.080 ± 5.394 (20.508-41.653)	45.086 ± 8.672 (28.088-62.083)	0.250	29.809 ± 6.484 (17.100-42.518)	37.487 ± 6.547 (24.655-50.318)	0.417	30.010 ± 8.956 (12.456-47.564)	35.717 ± 5.037 (25.846-45.589)	0.455
**ALT**	34.705 ± 5.072 (24.765-44.646)	30.786 ± 8.514 (14.098-47.474)	0.993	26.943 ± 5.991 (15.200-38.685)	43.085 ± 6.512 (30.321-55.848)	0.092	24.809 ± 7.181 (10.735-38.884)	38.897 ± 5.370 (28.371-49.422)	0.262
**Albumin**	34.746 ± 4.876 (25.188-44.304)	34.686 ± 11.024 (13.078-56.294)	0.754	35.715 ± 5.842 (23.724-46.626)	34.034 ± 7.364 (19.601-48.467)	0.721	33.000 ± 7.739 (17.831-48.169)	35.492 ± 5.593 (24.535-46.459)	0.949
**ALP**	35.274 ± 5.172 (2.138-45.411)	29.473 ± 8.641 (12.537-46.409)	0.833	30.133 ± 5.843 (18.681-41.585)	39.825 ± 7.266 (25.584-54.067)	0.167	28.091 ± 8.497 (11.437-44.745)	37.253 ± 5.247 (26.968-47.538)	0.272
**Bilirubin**	30.526 ± 4.574 (19.287-41.765)	44.482 ± 6.898 (30.965-57.998)	0.168	30.251 ± 6.629 (17.259-43.242)	38.150 ± 6.156 (26.084-50.216)	0.536	24.190 ± 4.574 (25.299-43.227)	36.914 ± 5.101 (26.916-46.913)	0.382

* Survival duration ± S.D. (95% CI)

** Units: Months

Supplementary Table S12. Kaplan-Meier survival analysis for multifocal recurrence of TACE-treated patients.

Biomarkers	Cut-off								
	Q1			Median			Q3		
	≥ Q1	< Q1	P-Value	≥ Q2	< Q2	P-Value	≥ Q3	< Q3	P-Value
**cfD$_{HCC}$ Score**	34.087 ± 5.230 (23.837-44.338)	44.167 ± 9.725 (25.105-63.228)	0.304	23.219 ± 5.512 (12.416-34.022)	54.015 ± 4.591 (45.018-63.013)	0.001	22.616 ± 9.369 (4.254-40.980)	41.531 ± 5.167 (31.404-51.658)	0.034
**cfDNA**	36.852 ± 5.194 (26.671-47.033)	21.500 ± 4.920 (11.856-31.144)	0.835	26.784 ± 5.920 (15.181-38.387)	48.284 ± 5.604 (37.301-59.267)	0.043	30.596 ± 10.096 (10.807-50.385)	39.227 ± 5.331 (28.779-49.675)	0.318
**cfAFP DNA**	35.999 ± 5.337 (25.538-46.460)	34.171 ± 7.920 (18.648-49.694)	0.897	35.063 ± 5.939 (23.423-26.703)	32.097 ± 6.938 (18.498-45.695)	0.749	33.857 ± 8.770 (16.668-51.046)	38.600 ± 5.625 (27.575-49.625)	0.743
**Total Protein**	33.725 ± 5.298 (23.341-44.109)	51.143 ± 7.375 (36.689-65.597)	0.330	32.938 ± 5.828 (21.515-44.361)	45.326 ± 6.059 (33.450-57.202)	0.595	17.861 ± 2.715 (12.540-23.182)	44.239 ± 5.083 (34.277-54.202)	0.070
**AFP**	18.500 ± 4.223 (10.223-26.777)	40.259 ± 5.591 (29.300-51.218)	0.147	29.755 ± 5.759 (18.467-41.044)	41.196 ± 6.829 (27.811-54.581)	0.218	31.772 ± 5.040 (21.893-41.650)	42.039 ± 8.296 (25.780-58.298)	0.364
**AST**	35.271 ± 5.802 (23.898-46.643)	39.794 ± 8.999 (22.155-57.432)	0.776	40.519 ± 6.989 (26.820-54.218)	32.627 ± 6.313 (20.254-45.000)	0.439	37.255 ± 8.423 (20.745-53.765)	39.946 ± 5.123 (26.906-46.986)	0.801
**ALT**	37.221 ± 5.277 (26.878-47.563)	30.786 ± 8.514 (14.098-47.474)	0.798	36.302 ± 6.791 (22.992-49.612)	37.407 ± 6.464 (24.739-50.076)	0.822	37.716 ± 9.105 (19.870-55.562)	36.356 ± 5.403 (25.767-46.946)	0.887
**Albumin**	37.267 ± 5.007 (27.454-47.081)	34.686 ± 11.024 (13.078-56.294)	0.639	35.024 ± 5.989 (23.286-46.763)	39.965 ± 7.004 (26.236-53.693)	0.892	35.018 ± 8.150 (19.045-50.992)	37.388 ± 5.678 (26.259-48.517)	0.996
**ALP**	42.538 ± 5.076 (32.589-52.487)	20.000 ± 6.627 (7.011-32.989)	0.0.053	38.113 ± 6.125 (26.109-50.117)	35.935 ± 7.020 (22.076-49.593)	0.910	30.768 ± 9.010 (13.109-48.427)	38.624 ± 5.394 (28.052-49.197)	0.440
**Bilirubin**	31.216 ± 4.690 (27.335-45.720)	48.741 ± 6.402 (36.192-61.289)	0.121	33.008 ± 7.085 (19.122-46.895)	39.652 ± 6.284 (27.336-51.968)	0.560	36.351 ± 9.453 (17.822-54.879)	37.276 ± 5.182 (27.118-47.433)	0.594

* Survival duration ± S.D. (95% CI)
** Units: Months

Supplementary Table S13. Kaplan-Meier survival analysis for OS of TACE-treated patients.

Biomarkers	Cut-off								
	Q1			Median			Q3		
	≥ Q1	< Q1	P-Value	≥ Q2	< Q2	P-Value	≥ Q3	< Q3	P-Value
**cfD$_{HCC}$ Score**	51.898 ± 4.400 (43.274-60.521)	68.300 ± 5.407 (57.701-78.899)	0.178	47.707 ± 35.593 (36.744-58.669)	65.665 ± 4.407 (57.027-74.303)	0.077	45.600 ± 8.690 (28.567-62.633)	61.098 ± 4.201 (52.865-69.332)	0.131
**cfDNA**	54.227 ± 4.146 (46.101-62.352)	61.587 ± 7.819 (46.262-76.262)	0.759	49.303 ± 5.241 (39.031-59.575)	64.685 ± 4.926 (55.030-74.341)	0.142	51.000 ± 8.224 (34.880-67.120)	58.955 ± 4.475 (50.185-67.726)	0.647
**cfAFP DNA**	58.348 ± 4.700 (49.136-67.559)	53.909 ± 7.289 (39.623-68.195)	0.981	58.252 ± 5.465 (47.540-68.964)	53.735 ± 5.706 (42.552-64.918)	0.870	62.424 ± 7.379 (47.961-76.887)	52.789 ± 4.426 (44.115-61.464)	0.430
**Total Protein**	57.969 ± 4.468 (49.212-66.726)	54.698 ± 8.249 (38.530-70.867)	0.989	59.110 ± 5.092 (49.129-69.090)	53.681 ± 5.850 (42.215-65.147)	0.788	56.241 ± 7.101 (42.323-70.159)	55.662 ± 4.375 (47.087-64.236)	0.693
**AFP**	32.700 ± 5.376 (22.163-43.237)	62.543 ± 4.164 (54.383-70.704)	0.032	48.680 ± 5.655 (37.640-59.720)	65.782 ± 4.410 (57.139-74.425)	0.084	55.327 ± 5.128 (45.276-65.379)	62.095 ± 3.962 (54.332-69.859)	0.277
**AST**	56.043 ± 4.703 (46.825-65.262;)	60.800 ± 6.440 (48.178-73.422)	0.323	50.860 ± 5.116 (40.833-60.886)	64.039 ± 5.246 (53.758-74.321)	0.212	53.800 ± 7.378 (39.339-68.261)	58.612 ± 4.618 (49.561-67.663)	0.837
**ALT**	57.675 ± 4.560 (48.738-66.613)	48.700 ± 6.957 (35.065-62.335)	0.856	49.104 ± 5.616 (38.097-60.111)	63.121 ± 4.954 (53.419-72.840)	0.223	48.500 ± 6.535 (35.692-61.308)	58.924 ± 4.545 (50.015-67.833)	0.723
**Albumin**	61.230 ± 4.197 (53.003-69.457)	43.557 ± 9.281 (25.367-61.747)	0.108	61.730 ± 4.772 (52.378-71.082)	50.134 ± 6.192 (37.997-62.271)	0.299	50.800 ± 5.606 (39.812-61.788)	59.480 ± 4.751 (50.167-68.793)	0.633
**ALP**	57.434 ± 4.657 (48.306-66.561)	52.943 ± 6.532 (40.140-65.745)	0.729	49.395 ± 5.264 (39.077-59.712)	64.068 ± 5.166 (53.943-74.194)	0.152	49.986 ± 8.026 (31.255-62.717)	60.693 ± 4.372 (52.124-69.263)	0.257
**Bilirubin**	N/A (All Cases Censored)			50.132 ± 5.262 (39.819-60.445)	64.187 ± 5.173 (54.048-74.325)	0.170	42.727 ± 7.653 (27.728-57.727)	63.807 ± 4.097 (55.777-71.836)	0.016

* Survival duration ± S.D. (95% CI)

** Units: Months

Supplementary Table S14. Univariate Cox regression analysis of the serum and plasma biomarkers for TACE-treated patients.

	Cut-off		cfD_{HCC} Score	cfDNA	cfAFP DNA	Total Protein	AFP	AST	ALT	Albumin	ALP	Bilirubin
Recurrence	Non-categorical		1.569<br>(1.201–2.048)<br>P = 0.001	1.111<br>(1.024–1.206)<br>P = 0.011	0.954<br>(0.871–1.045)<br>P = 0.314	1.224<br>(0.822–1.823)<br>P = 0.319	1.000<br>(1.000–1.000)<br>P = 0.017	1.006<br>(0.997–1.015)<br>P = 0.187	0.994<br>(0.977–1.011)<br>P = 0.495	0.924<br>(0.441–1.936)<br>P = 0.834	1.002<br>(0.998–1.005)<br>P = 0.370	1.076<br>(0.810–1.430)<br>P = 0.370
	Categorical	Q1	0.907<br>(0.423–1.946)<br>P = 0.801	0.689<br>(0.308–1.546)<br>P = 0.367	0.648<br>(0.305–1.373)<br>P = 0.257	1.912<br>(0.715–5.116)<br>P = 0.197	1.206<br>(0.551–2.637)<br>P = 0.639	1.330<br>(0.598–2.956)<br>P = 0.485	0.668<br>(0.299–1.492)<br>P = 0.325	0.700<br>(0.301–1.628)<br>P = 0.407	0.875<br>(0.392–1.955)<br>P = 0.745	2.072<br>(0.924–4.645)<br>P = 0.745
		Med	1.875<br>(0.943–3.728)<br>P = 0.073	1.619<br>(0.814–3.222)<br>P = 0.169	0.502<br>(0.243–1.038)<br>P = 0.063	0.998<br>(0.497–2.004)<br>P = 0.995	1.642<br>(0.813–3.319)<br>P = 0.167	0.953<br>(0.482–1.883)<br>P = 0.889	0.966<br>(0.489–1.905)<br>P = 0.920	0.921<br>(0.458–1.855)<br>P = 0.819	1.044<br>(0.529–2.059)<br>P = 0.901	1.443<br>(0.729–2.857)<br>P = 0.901
		Q3	2.115<br>(1.021–4.381)<br>P = 0.044	1.577<br>(0.765–3.251)<br>P = 0.217	1.003<br>(0.478–2.105)<br>P = 0.994	1.610<br>(0.767–3.381)<br>P = 0.208	1.662<br>(0.74–3.73)<br>P = 0.218	1.877<br>(0.871–4.045)<br>P = 0.108	0.871 (0.405–<br>1.871) P =<br>0.723	0.848<br>(0.404–1.781)<br>P = 0.663	1.061<br>(0.492–2.292)<br>P = 0.879	1.605<br>(0.762–3.384)<br>P = 0.879
Marginal Recurrence	Non-categorical		1.477<br>(0.974–2.240)<br>P = 0.066	1.058<br>(0.901–1.242)<br>P = 0.492	0.903<br>(0.779–1.047)<br>P = 0.175	1.883<br>(0.85–4.17)<br>P = 0.119	1.000<br>(1.000–1.000)<br>P = 0.033	1.004<br>(0.99–1.017)<br>P = 0.588	1.007<br>(0.987–1.027)<br>P = 0.489	1.036<br>(0.366–2.932)<br>P = 0.947	1.001<br>(0.996–1.006)<br>P = 0.571	1.001<br>(0.654–1.532)<br>P = 0.571
	Categorical	Q1	1.542<br>(0.443–5.374)<br>P = 0.496	0.740<br>(0.238–2.297)<br>P = 0.602	0.936<br>(0.302–2.905)<br>P = 0.909	2.336<br>(0.505–10.812)<br>P = 0.278	1.779<br>(0.642–4.927)<br>P = 0.268	2.026<br>(0.578–7.108)<br>P = 0.270	0.994<br>(0.282–3.503)<br>P = 0.993	0.820<br>(0.230–2.922)<br>P = 0.760	0.886<br>(0.282–2.784)<br>P = 0.836	2.162<br>(0.688–6.793)<br>P = 0.836
		Med	2.471<br>(0.908–6.72)<br>P = 0.076	2.128<br>(0.782–5.790)<br>P = 0.139	0.435<br>(0.156–1.213)<br>P = 0.112	1.543<br>(0.537–4.435)<br>P = 0.421	2.380<br>(0.822–6.894)<br>P = 0.110	1.479<br>(0.558–3.918)<br>P = 0.431	2.366<br>(0.824–6.791)<br>P = 0.109	0.840<br>(0.315–2.237)<br>P = 0.727	2.026<br>(0.712–5.770)<br>P = 0.186	1.343<br>(0.514–3.508)<br>P= 0.186
		Q3	1.874<br>(0.649–5.404)<br>P = 0.245	1.100<br>(0.357–3.39)<br>P = 0.868	0.548<br>(0.157–1.911)<br>P = 0.345	2.419<br>(0.895–6.538)<br>P = 0.082	2.017<br>(0.574–7.087)<br>P = 0.274	1.539<br>(0.478–4.958)<br>P = 0.470	1.707<br>(0.648–4.496)<br>P = 0.279	1.032<br>(0.379–2.808)<br>P = 0.951	1.726<br>(0.629–4.733)<br>P = 0.289	1.576<br>(0.550–4.516)<br>P = 0.289
Multifocal Recurrence	Non-categorical		1.728<br>(1.183–2.525)<br>P = 0.005	1.126<br>(0.999–1.268)<br>P = 0.052	1.002<br>(0.885–1.134)<br>P = 0.976	1.779<br>(0.779–4.061)<br>P = 0.171	1.000<br>(1.000–1.000)<br>P = 0.021	1.000<br>(0.985–1.016)<br>P = 0.988	0.996<br>(0.972–1.022)<br>P = 0.781	1.026<br>(0.339–3.108)<br>P = 0.963	1.000<br>(0.994–1.006)<br>P = 0.985	1.017<br>(0.655–1.578)<br>P = 0.985
	Categorical	Q1	2.138<br>(0.481–9.493)<br>P = 0.318	0.866<br>(0.241–3.118)<br>P = 0.826	1.088<br>(0.302–3.918)<br>P = 0.898	2.126<br>(0.45–10.037)<br>P = 0.341	2.161<br>(0.741–6.304)<br>P = 0.158	1.182<br>(0.372–3.752)<br>P = 0.777	0.847<br>(0.235–3.048)<br>P = 0.799	0.736<br>(0.203–2.677)<br>P = 0.642	0.361<br>(0.123–1.065)<br>P = 0.065	2.673<br>(0.733–9.741)<br>P = 0.065
		Med	8.544<br>(1.919–38.042)<br>P = 0.005	3.083<br>(0.976–9.742)<br>P = 0.055	1.200<br>(0.391–3.686)<br>P = 0.750	1.340<br>(0.452–3.975)<br>P = 0.598	1.966<br>(0.655–5.903)<br>P = 0.228	0.665<br>(0.235–1.888)<br>P = 0.444	1.125<br>(0.402–3.149)<br>P = 0.823	0.930<br>(0.326–2.654)<br>P = 0.893	0.943<br>(0.341–2.613)<br>P = 0.911	1.352<br>(0.486–3.76)<br>P = 0.911
		Q3	2.961<br>(1.032–8.49)<br>P = 0.043	1.718<br>(0.584–5.055)<br>P = 0.326	1.197<br>(0.406–3.526)<br>P = 0.745	2.593<br>(0.887–7.580)<br>P = 0.082	1.789<br>(0.498–6.431)<br>P = 0.0373	1.183<br>(0.319–4.392)<br>P = 0.802	0.921<br>(0.292–2.903)<br>P = 0.888	0.997<br>(0.339–2.932)<br>P = 0.996	1.526<br>(0.515–4.518)<br>P = 0.445	1.366<br>(0.429–4.347)<br>P = 0.445
Survival	Non-categorical		2.443<br>(1.517–3.932)<br>P < 0.0001	1.199<br>(1.057–1.359)<br>P = 0.005	0.895<br>(0.735–1.091)<br>P = 0.273	1.184<br>(0.589–2.38)<br>P = 0.635	1.000<br>(1.000–1.000)<br>P = 0.002	1.004<br>(0.991–1.018)<br>P = 0.546	1.004<br>(0.974–1.036)<br>P = 0.782	0.618<br>(0.183–2.09)<br>P = 0.439	1.001<br>(0.996–1.006)<br>P = 0.630	1.290<br>(0.813–2.046)<br>P = 0.630
	Categorical	Q1	3.733<br>(0.476–29.243)<br>P = 0.210	1.271<br>(0.274–5.896)<br>P = 0.759	0.984<br>(0.26–3.719)<br>P = 0.981	1.011<br>(0.218–4.691)<br>P = 0.989	3.556<br>(1.028–12.296)<br>P = 0.045	2.707<br>(0.346–21.187)<br>P = 0.343	1.152<br>(0.248–5.344)<br>P = 0.857	0.377<br>(0.109–1.299)<br>P = 0.122	1.310<br>(0.283–6.069)<br>P = 0.730	36.045<br>(0.156–8336.5)<br>P = 0.730
		Med	3.123<br>(0.826–11.814)<br>P = 0.093	2.606<br>(0.691–9.834)<br>P = 0.157	0.905<br>(0.274–2.989)<br>P = 0.870	0.849<br>(0.258–2.793)<br>P = 0.788	3.074<br>(0.806–11.722)<br>P = 0.100	2.277<br>(0.603–8.593)<br>P = 0.225	2.124<br>(0.615–7.339) P<br>= 0.234	0.536<br>(0.162–1.77)<br>P = 0.306	2.546<br>(0.675–9.601)<br>P = 0.168	2.457<br>(0.651–9.268)<br>P = 0.168
		Q3	2.503<br>(0.73–8.587)<br>P = 0.145	1.363<br>(0.361–5.14)<br>P = 0.648	0.544<br>(0.117–2.531)<br>P = 0.437	1.281<br>(0.374–4.381)<br>P = 0.694	2.300<br>(0.492–10.759)<br>P = 0.290	1.150<br>(0.304–4.346)<br>P = 0.837	1.273<br>(0.334–4.846)<br>P = 0.723	1.314<br>(0.383–4.501)<br>P = 0.664	2.011<br>(0.586–6.902)<br>P = 0.267	3.912<br>(1.188–12.885)<br>P = 0.267

* Hazard ratio (95%CI)

Supplementary Table S15. Kaplan-Meier survival analysis for RFS of non-TACE patients.

Biomarkers	Cut-off								
	Q1			Median			Q3		
	≥ Q1	< Q1	P-Value	≥ Q2	< Q2	P-Value	≥ Q3	< Q3	P-Value
**cfD$_{HCC}$ Score**	60.433 ± 5.587 (49.483-71.384)	38.947 ± 4.625 (29.882-48.012)	0.385	54.597 ± 6.440 (41.974-67.219)	61.264 ± 6.735 (48.064-74.465)	0.398	56.198 ± 9.386 (37.803-74.594)	58.893 ± 5.941 (47.249-70.537)	0.931
**cfDNA**	60.893 ± 5.589 (49.937-71.848)	37.460 ± 4.655 (28.335-46.584)	0.264	53.911 ± 6.171 (41.815-66.007)	61.139 ± 7.372 (46.689-75.588)	0.323	51.441 ± 9.534 (32.755-70.127)	60.143 ± 5.956 (48.468-71.817)	0.458
**cfAFP DNA**	53.937 ± 5.601 (42.958-64.916)	69.902 ± 8.322 (53.590-86.214)	0.157	52.426 ± 5.685 (41.284-63.568)	62.824 ± 8.209 (46.734-78.915)	0.347	59.557 ± 6.913 (46.008-73.107)	57.859 ± 7.065 (44.011-71.708)	0.615
**Total Protein**	55.736 ± 4.720 (46.486-64.986)	60.471 ± 10.581 (39.732-81.210)	0.986	54.757 ± 5.815 (43.360-66.154)	61.180 ± 7.272 (46.928-75.432)	0.959	36.482 ± 4.181 (28.287-44.676)	61.048 ± 5.593 (50.084-72.011)	0.270
**AFP**	59.249 ± 6.860 (45.804-72.694)	60.631 ± 7.543 (45.846-75.415)	0.623	52.693 ± 6.505 (39.943-65.442)	62.609 ± 7.047 (48.797-76.422)	0.344	62.236 ± 9.389 (44.833-81.639)	57.849 ± 5.822 (46.438-69.259)	0.715
**AST**	54.503 ± 5.582 (43.563-65.443)	61.081 ± 5.291 (50.710-71.452)	0.062	54.678 ± 6.773 (41.403-67.953)	53.175 ± 4.218 (44.908-61.442)	0.266	50.158 ± 8.521 (33.456-66.859)	61.638 ± 6.612 (48.680-74.597)	0.314
**ALT**	59.304 ± 5.799 (47.939-70.670)	50.761 ± 5.930 (39.137-62.384)	0.998	63.159 ± 7.110 (49.222-77.095)	48.195 ± 4.211 (39.941-56.449)	0.559	54.860 ± 8.496 (38.209-71.512)	59.357 ± 6.448 (46.720-71.995)	0.636
**Albumin**	55.294 ± 4.727 (46.029-64.559)	56.451 ± 11.045 (34.803-78.100)	0.870	59.576 ± 5.727 (48.351-70.801)	55.374 ± 7.252 (41.160-69.589)	0.259	58.031 ± 7.057 (44.199-71.862)	59.681 ± 6.442 (47.054-72.308)	0.630
**ALP**	52.664 ± 4.852 (43.155-62.173)	74.856 ± 11.338 (52.633-97.079)	0.141	48.770 ± 4.937 (39.094-58.446)	66.802 ± 7.135 (52.818-80.787)	0.228	34.430 ± 4.409 (25.788-43.073)	62.426 ± 5.607 (51.436-73.416)	0.071
**Bilirubin**	53.430 ± 45.167 (43.302-63.558)	70.391 ± 10.693 (49.432-91.349)	0.302	54.302 ± 6.220 (42.112-68.324)	64.186 ± 8.006 (48.494-79.878)	0.580	53.451 ± 8.886 (36.034-70.867)	59.492 ± 6.178 (47.384-71.600)	0.783

* Survival duration ± S.D. (95% CI)

** Units: Months

Supplementary Table S16. Kaplan-Meier survival analysis for marginal recurrence of non-TACE patients.

Biomarkers	Cut-off								
	Q1			Median			Q3		
	≥ Q1	< Q1	P-Value	≥ Q2	< Q2	P-Value	≥ Q3	< Q3	P-Value
**cfD$_{HCC}$ Score**	98.823 ± 7.849 (84.440-114.206)	59.680 ± 2.273 (55.225-64.135)	0.686	90.257 ± 6.013 (78.472-102.043)	108.274 ± 8.595 (91.428-125.119)	0.331	84.636 ± 10.221 (64.603-104.669)	109.964 ± 7.303 (95.380-124.008)	0.131
**cfDNA**	98.999 ± 7.829 (83.654-114.343)	59.583 ± 2.366 (54.946-64.220)	0.746	90.772 ± 5.684 (79.632-101.911)	105.879 ± 10.194 (85.898-125.860)	0.422	82.851 ± 11.427 (60.454-105.249)	109.719 ± 7.300 (95.412-124.027)	0.114
**cfAFP DNA**	97.450 ± 8.173 (81.430-113.470)	93.227 ± 3.686 (86.003-100.452)	0.479	94.850 ± 5.447 (84.173-105.526)	100.818 ± 10.260 (80.709-120.927)	0.454	95.800 ± 6.977 (82.126-109.474)	102.449 ± 9.977 (82.894-122.003)	0.539
**Total Protein**	88.530 ± 3.936 (80.836-96.224)	100.524 ± 9.678 (81.555-119.492)	0.822	86.253 ± 5.345 (75.777-96.728)	103.462 ± 8.061 (87.662-119.262)	0.653	55.750 ± 2.203 (51.433-60.067)	99.193 ± 7.809 (83.889-114.498)	0.814
**AFP**	99.430 ± 9.580 (80.653-118.207)	81.936 ± 6.914 (68.384-95.488)	0.136	90.157 ± 8.826 (72.858-107.457)	103.398 ± 6.027 (96.585-120.211)	0.640	101.000 (101.000)	104.770 ± 6.965 (91.118-118.421)	0.381
**AST**	97.187 ± 8.298 (80.923-113.452)	76.292 ± 2.651 (71.095-81.488)	0.448	104.977 ± 8.167 (88.970-120.984)	72.164 ± 2.825 (66.626-77.701)	0.269	91.167 ± 8.007 (75.474-106.860)	108.220 ± 7.339 (93.836-122.604)	0.514
**ALT**	98.716 ± 8.289 (82.470-114.962)	73.274 ± 3.884 (65.661-80.887)	0.840	106.754 ± 7.817 (91.433-122.075)	70.728 ± 3.537 (63.796-77.660)	0.223	N/A (All Cases Censored)		
**Albumin**	87.105 ± 4.554 78.179-96.032)	97.467 ± 11.224 (75.457-119.466)	0.655	89.403 ± 4.387 (80.804-98.001)	99.966 ± 8.453 (83.398-116.533)	0.469	91.500 ± 4.763 (82.164-100.836)	100.382 ± 7.857 (84.982-115.782)	0.259
**ALP**	90.223 ± 5.338 (79.761-100.685)	111.791 ± 6.889 (98.288-125.295)	0.780	79.475 ± 5.182 (69.318-89.631)	106.761 ± 7.798 (91.476-122.046)	0.241	50.086 ± 5.037 (40.214-59.958)	104.163 ± 7.794 (88.887-119.439)	0.002
**Bilirubin**	94.349 ± 3.773 (86.955-101.743)	103.274 ± 9.920 (83.831-122.717)	0.796	91.543 ± 5.073 (81.600-101.483)	108.274 ± 8.595 (84.530-114.676)	0.391	92.428 ± 7.017 (78.675-106.181)	108.101 ± 7.352 (93.962-122.511)	0.575

* Survival duration ± S.D. (95% CI)

** Units: Months

Supplementary Table S17. Kaplan-Meier survival analysis for multifocal recurrence of non-TACE patients.

Biomarkers	Cut-off								
	Q1			Median			Q3		
	≥ Q1	< Q1	P-Value	≥ Q2	< Q2	P-Value	≥ Q3	< Q3	P-Value
**cfD$_{HCC}$ Score**	98.365 ± 7.287 (84.082-112.648)	49.652 ± 4.420 (40.988-58.315)	0.254	85.829 ± 5.248 (75.543-96.115)	91.630 ± 9.025 (73.942-109.318)	0.690	88.534 ± 6.700 (75.402-101.667)	91.219 ± 8.477 (74.604-107.835)	0.554
**cfDNA**	98.505 ± 7.273 (84.251-112.760)	49.120 ± 4.579 (40.145-58.094)	0.214	82.769 ± 5.328 (72.317-93.202)	103.472 ± 5.989 (91.734-115.210)	0.742	82.851 ± 6.700 (75.402-101.667)	91.468 ± 8.467 (74.873-108.063)	0.605
**cfAFP DNA**	104.347 ± 4.903 (94.747-113.956)	79.078 ± 7.750 (63.887-94.269)	0.387	88.113 ± 4.519 (79.225-96.970)	88.224 ± 9.275 (70.045-106.403)	0.349	97.593 ± 3.344 (91.039-104.146)	86.451 ± 8.508 (69.776-103.126)	0.023
**Total Protein**	81.431 ± 4.052 (73.489-89.372)	106.696 ± 9.240 (90.545-122.846)	0.510	84.440 ± 4.702 (75.224-93.656)	98.965 ± 6.870 (85.501-112.430)	0.743	49.408 ± 3.895 (41.773-57.042)	96.085 ± 7.274 (81.828-110.342)	0.854
**AFP**	98.758 ± 5.549 (87.882-109.634)	90.126 ± 5.713 (78.928-101.325)	0.322	78.126 ± 5.985 (66.395-89.857)	99.353 ± 9.019 (81.676-117.031)	0.097	78.389 ± 8.028 (62.655-94.123)	94.992 ± 8.126 (78.126-111.857)	0.320
**AST**	90.233 ± 7.457 (75.618-104.848)	76.346 ± 2.602 (71.246-81.447)	0.587	93.457 ± 8.424 (76.946-109.968)	68.321 ± 3.473 (61.513-75.129)	0.646	83.611 ± 7.828 (68.268-98.955)	92.964 ± 8.527 (76.252-109.676)	0.903
**ALT**	93.774 ± 7.370 (79.328-108.219)	70.880 ± 4.399 (62.259-79.501)	0.458	89.996 ± 8.381 (73.569-106.422)	70.679 ± 3.183 (64.441-76.918)	0.189	77.583 ± 8.414 (61.093-94.074)	104.296 ± 4.907 (94.678-113.913)	0.287
**Albumin**	83.424 ± 3.751 (76.072-90.776)	97.467 ± 12.056 (70.641-117.902)	0.659	87.254 ± 4.059 (79.299-95.210)	93.490 ± 7.783 (78.235-108.746)	0.190	85.761 ± 5.870 (45.280-62.329)	99.876 ± 5.743 (88.619-111.132)	0.960
**ALP**	81.571 ± 4.337 (73.071-90.070)	110.959 ± 7.387 (96.481-125.438)	0.203	79.805 ± 4.259 (71.456-88.153)	93.096 ± 8.183 (77.058-109.134)	0.571	53.805 ± 4.349 (45.280-62.329)	95.530 ± 7.206 (81.405-109.654)	0.960
**Bilirubin**	85.760 ± 4.044 (77.834-93.686)	94.318 ± 9.843 (75.027-113.610)	0.386	84.696 ± 5.098 (74.703-94.689)	101.284 ± 6.255 (89.024-115.543)	0.912	80.974 ± 7.905 (65.480-96.468)	93.882 ± 8.582 (77.061-110.703)	0.628

* Survival duration ± S.D. (95% CI)

** Units: Months

Supplementary Table S18. Kaplan-Meier survival analysis for OS of non-TACE patients.

Biomarkers	Cut-off								
	Q1			Median			Q3		
	≥ Q1	< Q1	P-Value	≥ Q2	< Q2	P-Value	≥ Q3	< Q3	P-Value
**cfD$_{HCC}$ Score**	120.383 ± 3.774 (112.986-127.780)	86.840 ± 4.379 (78.258-95.422)	0.807	97.682 ± 4.725 (88.420-106.943)	122.107 ± 5.619 (111.093-133.121)	0.119	83.864 ± 7.645 (68.881-98.847)	83.864 ± 7.645 (114.732-131.343)	0.007
**cfDNA**	120.521 ± 3.721 (113.227-127.815)	86.708 ± 4.557 (77.776-95.640)	0.750	98.015 ± 4.604 (88.992-107.038)	121.131 ± 6.353 (108.680-133.582)	0.153	81.00 ± 7.882 (65.552-96.448)	124.068 ± 4.392 (115.459-132.676)	0.001
**cfAFP DNA**	117.922 ± 4.869 (108.379-127.466)	95.679 ± 4.982 (85.913-105.444)	0.970	100.222 ± 4.115 (92.157-108.288)	122.080 ± 4.450 (113.359-130.801)	0.618	100.082 ± 4.110 (92.027-108.136)	119.231 ± 4.281 (110.841-127.621)	0.612
**Total Protein**	118.342 ± 5.787 (107.000-129.684)	113.120 ± 8.035 (97.372-128.868)	0.458	117.743 ± 7.499 (103.044-132.442)	115.689 ± 5.217 (105.463-125.915)	0.482	83.891 ± 5.100 (73.895-93.888)	121.789 ± 3.552 (114.827-128.750)	0.294
**AFP**	114.843 ± 6.080 (102.926-126.759)	115.815 ± 4.107 (107.765-123.864)	0.339	114.843 ± 6.080 (102.926-126.759)	122.774 ± 3.637 (115.645-129.902)	0.339	91.966 ± 5.985 (80.236-103.697)	119.895 ± 4.542 (110.994-128.796)	0.307
**AST**	N/A (All Cases Censored)			113.517 ± 5.553 (102.634-124.400)	114.673 ± 10.862 (93.383-135.963)	0.189	90.959 ± 7.694 (75.879-106.039)	120.332 ± 6.266 (108.049-132.614)	0.0025
**ALT**	116.790 ± 4.911 (107.165-126.416; P = 0.464)	115.963 ± 3.962 (108.198-123.728)	0.464	116.167 ± 5.038 (106.292-126.042)	117.591 ± 7.492 (102.906-132.276)	0.508	102.250 ± 5.281 (91.900-112.600)	116.195 ± 6.003 (104.428-127.961)	0.854
**Albumin**	118.623 ± 5.370 (107.392-129.854)	110.567 ± 9.298 (92.343-128.792)	0.318	118.000 ± 7.451 (103.395-132.605)	114.801 ± 5.558 (103.907-125.695)	0.386	118.333 ± 7.822 (103.003-133.664)	117.344 ± 4.173 (109.165-125.523)	0.633
**ALP**	118.418 ± 4.831 (108.950-127.886)	118.247 ± 6.584 (105.343-131.151)	0.946	118.418 ± 5.720 (107.340-129.763)	117.725 ± 4.919 (108.083-127.366)	0.834	84.244 ± 5.913 (72.655-95.833)	123.660 ± 3.088 (117.608-129.712)	0.047
**Bilirubin**	107.623 ± 4.660 (98.489-116.756)	125.240 ± 4.664 (116.099-134.381)	0.387	104.321 ± 4.245 (110.001-126.641)	125.365 ± 3.217 (119.059-131.671)	0.169	106.847 ± 7.135 (92.863-120.831)	118.980 ± 5.091 (109.001-128.958)	0.427

* Survival duration ± S.D. (95% CI)

** Units: Months

Supplementary Table S19. Univariate Cox regression analysis of the serum and plasma biomarkers for non-TACE patients.

HR(95%CI)	Cut-off		cfD_{HCC} Score	cfDNA	cfAFP DNA	Total Protein	AFP	AST	ALT	Albumin	ALP	Bilirubin
Recurrence	Non-categorical		0.923<br>(0.692–1.231)<br>P = 0.586	0.981<br>(0.909–1.059)<br>P = 0.622	0.987<br>(0.913–1.068)<br>P = 0.749	1.091<br>(0.825–1.443)<br>P = 0.540	1.000<br>(0.999–1.000)<br>P = 0.414	1.001<br>(0.998–1.004)<br>P = 0.527	1.000<br>(0.996–1.004)<br>P = 0.961	0.938<br>(0.581–1.515)<br>P = 0.794	1.012<br>(1.004–1.02)<br>P = 0.004	0.979<br>(0.688–1.392)<br>P = 0.004
	Categorical	Q1	0.746<br>(0.381–1.457)<br>P = 0.390	0.685<br>(0.350–1.343)<br>P = 0.271	1.678<br>(0.809–3.48)<br>P = 0.164	1.006<br>(0.518–1.955)<br>P = 0.986	1.173<br>(0.618–2.228)<br>P = 0.625	2.029<br>(0.944–4.358)<br>P = 0.070	0.997<br>(0.516–1.928)<br>P = 0.993	0.944<br>(0.469–1.898)<br>P = 0.871	1.710<br>(0.826–3.542)<br>P = 0.149	1.428<br>(0.72–2.833)<br>P = 0.149
		Med	1.271<br>(0.725–2.229)<br>P = 0.403	1.326<br>(0.754–2.332)<br>P = 0.328	1.313<br>(0.740–2.328)<br>P = 0.352	1.015<br>(0.574–1.795)<br>P = 0.959	1.309<br>(0.746–2.296)<br>P = 0.349	1.387<br>(0.774–2.487)<br>P = 0.272	0.841<br>(0.468–1.512)<br>P = 0.563	0.723<br>(0.408–1.278)<br>P = 0.264	1.424<br>(0.795–2.551)<br>P = 0.234	1.171<br>(0.666–2.059)<br>P = 0.234
		Q3	1.029<br>(0.535–1.98)<br>P = 0.931	1.271<br>(0.671–2.409)<br>P = 0.462	0.857<br>(0.468–1.570)<br>P = 0.617	1.452<br>(0.741–2.844)<br>P = 0.277	0.883<br>(0.449–1.733)<br>P = 0.717	1.365<br>(0.74–2.52)<br>P = 0.319	1.163<br>(0.619–2.182)<br>P = 0.639	0.861<br>(0.467–1.589)<br>P = 0.633	1.826<br>(0.935–3.566)<br>P = 0.078	1.091<br>(0.584–2.038)<br>P = 0.078
Marginal Recurrence	Non-categorical		1.423<br>(0.919–2.202)<br>P = 0.113	1.101<br>(0.981–1.235)<br>P = 0.103	0.979<br>(0.782–1.225)<br>P = 0.853	0.955<br>(0.490–1.863)<br>P = 0.894	0.997<br>(0.990–1.005)<br>P = 0.483	0.999<br>(0.99–1.009)<br>P = 0.883	0.986<br>(0.964–1.009)<br>P = 0.230	0.639<br>(0.184–2.218)<br>P = 0.480	1.023<br>(1.004–1.042)<br>P = 0.018	1.096<br>(0.462–2.597)<br>P = 0.018
	Categorical	Q1	1.551<br>(0.180–13.375)<br>P = 0.690	1.424<br>(0.164–12.337)<br>P = 0.749	2.120<br>(0.251–17.945)<br>P = 0.490	0.828<br>(0.159–4.309)<br>P = 0.823	0.333<br>(0.073–1.518)<br>P = 0.156	2.216<br>(0.266–18.451)<br>P = 0.462	0.845<br>(0.163–4.373)<br>P = 0.840	0.684<br>(0.128–3.669)<br>P = 0.658	1.260<br>(0.246–6.449)<br>P = 0.781	0.821<br>(0.184–3.668)<br>P = 0.781
		Med	2.007<br>(0.477–8.444)<br>P = 0.342	1.780<br>(0.425–7.459)<br>P = 0.430	0.583<br>(0.139–2.446)<br>P = .461	1.409<br>(0.312–6.352)<br>P = 0.656	0.712<br>(0.17–2.988)<br>P = 0.642	0.407<br>(0.078–2.122)<br>P = 0.286	0.372<br>(0.071–1.951)<br>P = 0.242	0.578<br>(0.128–2.605)<br>P = 0.475	2.575<br>(0.497–13.345)<br>P = 0.260	1.857<br>(0.441–7.825)<br>P = 0.260
		Q3	2.834<br>(0.692–11.607)<br>P = 0.147	2.970<br>(0.722–12.209)<br>P = 0.131	0.604<br>(0.119–3.07)<br>P = 0.544	0.772<br>(0.088–6.742)<br>P = 0.815	0.398<br>(0.047–3.363)<br>P = 0.397	1.620<br>(0.375–6.995)<br>P = 0.518	0.029<br>(0–34.024)<br>P = 0.327	0.313<br>(0.037–2.639)<br>P = 0.286	9.004<br>(1.621–50.009)<br>P = 0.012	1.515<br>(0.350–6.564)<br>P = 0.012
Multifocal Recurrence	Non-categorical		0.746<br>(0.378–1.475)<br>P = 0.400	0.932<br>(0.781–1.111)<br>P = 0.431	0.769<br>(0.597–0.992)<br>P = 0.043	1.084<br>(0.681–1.726)<br>P = 0.732	0.999<br>(0.996–1.001)<br>P = 0.339	1.002<br>(0.997–1.006)<br>P = 0.489	1.001<br>(0.995–1.007)<br>P = 0.679	0.599<br>(0.258–1.389)<br>P = 0.232	1.004<br>(0.989–1.019)<br>P = 0.600	1.075<br>(0.603–1.916)<br>P = 0.600
	Categorical	Q1	0.560<br>(0.203–1.542)<br>P = 0.262	0.533<br>(0.194–1.466)<br>P = 0.223	0.647<br>(0.239–1.752)<br>P = 0.391	1.537<br>(0.423–5.585)<br>P = 0.514	1.857<br>(0.533–6.47)<br>P = 0.331	6.338<br>(0.837–48.011)<br>P = 0.074	1.599<br>(0.456–5.613)<br>P = 0.464	0.769<br>(0.239–2.479)<br>P = 0.660	2.520<br>(0.575–11.031)<br>P = 0.220	0.636<br>(0.235–1.722)<br>P = 0.220
		Med	0.822<br>(0.312–2.165)<br>P = 0.692	1.174<br>(0.451–3.054)<br>P = 0.743	0.632<br>(0.24–1.669)<br>P = 0.355	0.853<br>(0.328–2.219)<br>P = 0.744	2.274<br>(0.838–6.170)<br>P = 0.107	1.257<br>(0.471–3.353)<br>P = 0.648	1.943<br>(0.706–5.348)<br>P = 0.199	0.529<br>(0.200–1.396)<br>P = 0.198	0.753<br>(0.280–2.022)<br>P = 0.573	0.948<br>(0.364–2.467)<br>P = 0.573
		Q3	0.688<br>(0.197–2.398)<br>P = 0.557	0.721<br>(0.207–2.515)<br>P = 0.608	0.135<br>(0.018–1.023)<br>P = 0.053	1.112<br>(0.359–3.448)<br>P = 0.854	1.648<br>(0.608–4.465)<br>P = 0.326	1.046<br>(0.340–3.218)<br>P = 0.937	1.706<br>(0.630–4.623)<br>P = 0.294	0.827<br>(0.291–2.347)<br>P = 0.721	0.969<br>(0.276–3.401)<br>P = 0.960	1.293<br>(0.455–3.680)<br>P = 0.960
Survival	Non-categorical		1.871<br>(1.384–2.528)<br>P < 0.0001	1.166<br>(1.073–1.267)<br>P < 0.0001	0.908<br>(0.689–1.198)<br>P = 0.496	0.819<br>(0.464–1.446)<br>P = 0.491	1.000<br>(1.000–1.000)<br>P = 0.001	1.005<br>(1.000–1.009)<br>P = 0.043	1.001<br>(0.992–1.010)<br>P = 0.768	0.643<br>(0.195–2.128)<br>P = 0.470	1.008<br>(1.004–1.012)<br>P < 0.0001	1.933<br>(1.164–3.212)<br>P < 0.0001
	Categorical	Q1	0.818<br>(0.162–4.13)<br>P = 0.808	0.770<br>(0.152–3.887)<br>P = 0.751	0.970<br>(0.192–4.905)<br>P = 0.970	0.583<br>(0.137–2.474)<br>P = 0.464	2.682<br>(0.327–22.019)<br>P = 0.358	31.989<br>(0.030–33687.4)<br>P = 0.329	2.154<br>(0.262–17.692)<br>P = 0.475	0.484<br>(0.113–2.074)<br>P = 0.329	0.946<br>(0.19–4.715)<br>P = 0.946	2.446<br>(0.300–19.939)<br>P = 0.946
		Med	3.317<br>(0.668–16.461)<br>P = 0.142	3.030<br>(0.611–15.012)<br>P = 0.175	1.438<br>(0.341–6.056)<br>P = 0.621	0.602<br>(0.143–2.526)<br>P = 0.488	3.276<br>(0.66–16.264)<br>P = 0.147	2.818<br>(0.561–14.157)<br>P = 0.208	1.615<br>(0.385–6.784)<br>P = 0.513	0.535<br>(0.127–2.252)<br>P = 0.394	0.862<br>(0.214–3.471)<br>P = 0.834	2.922<br>(0.588–14.512)<br>P = 0.832
		Q3	5.783<br>(1.377–24.296)<br>P = 0.017	9.573<br>(1.930–47.470)<br>P = 0.006	0.656<br>(0.127–3.387)<br>P = 0.615	2.138<br>(0.499–9.163)<br>P = 0.306	2.094<br>(0.49–8.948)<br>P = 0.318	4.585<br>(1.074–19.579)<br>P = 0.040	0.860<br>(0.171–4.309)<br>P = 0.854	0.678<br>(0.136–3.374)<br>P = 0.635	3.734<br>(0.923–15.107)<br>P = 0.065	1.773<br>(0.423–7.437)<br>P = 0.065

* Hazard ratio (95%CI)

Supplementary Table S20. Univariate Cox regression analysis of the serum and plasma biomarkers for all patients.

	Cut-off		cfD$_{HCC}$ Score	cfDNA	cfAFP DNA	Total Protein	AFP	AST	ALT	Albumin	ALP	Bilirubin
Recurrence	Non-categorical		1.081<br>(0.884–1.321)<br>P = 0.447	1.005<br>(0.949–1.065)<br>P = 0.854	1.001<br>(0.947–1.058)<br>P = 0.964	1.226<br>(0.981–1.533)<br>P = 0.073	1.000<br>(1.000–1.000)<br>P = 0.002	1.000<br>(0.998–1.003)<br>P = 0.904	0.997<br>(0.994–1.001)<br>P = 0.202	0.923<br>(0.627–1.359)<br>P = 0.686	1.005<br>(1.003–1.008)<br>P < 0.001	1.099<br>(0.875–1.381)<br>P = 0.416
	Categorical	Q1	0.930<br>(0.558–1.547)<br>P = 0.779	0.809<br>(0.484–1.349)<br>P = 0.416	1.754<br>(1.010–3.045)<br>P = 0.046	1.393<br>(0.799–2.426)<br>P = 0.242	1.253<br>(0.767–2.048)<br>P = 0.368	1.681<br>(0.957–2.954)<br>P = 0.071	0.763<br>(0.470–1.240)<br>P = 0.275	0.881<br>(0.514–1.510)<br>P = 0.881	1.906<br>(1.071–3.391)<br>P = 0.028	(1.472<br>(0.877–2.471)<br>P = 0.144
		Med	1.406<br>(0.913–2.166)<br>P = 0.122	1.346<br>(0.873–2.078)<br>P = 0.179	1.126<br>(0.729–1.738)<br>P = 0.594	1.178<br>(0.758–1.828)<br>P = 0.467	1.303<br>(0.847–2.007)<br>P = 0.229	1.134<br>(0.729–1.761)<br>P = 0.577	0.712<br>(0.457–1.109)<br>P = 0.133	0.793<br>(0.513–1.226)<br>P = 0.298	1.905<br>(1.223–2.968)<br>P = 0.004	1.235<br>(0.800–1.906)<br>P = 0.341
		Q3	1.175<br>(0.715–1.931)<br>P = 0.524	1.185<br>(0.714–1.966)<br>P = 0.512	1.038<br>(0.651–1.655)<br>P = 0.876	1.862<br>(1.155–3.000)<br>P = 0.011	1.253<br>(0.767–2.048)<br>P = 0.368	1.213<br>(0.748–1.965)<br>P = 0.434	0.746<br>(0.445–1.249)<br>P = 0.264	0.851<br>(0.531–1.363)<br>P = 0.501	2.485<br>(1.536–4.018)<br>P = 0.001	1.201<br>(0.744–1.937)<br>P = 0.454
Marginal Recurrence	Non-categorical		1.277<br>(0.944–1.726)<br>P = 0.113	1.041<br>(0.951–1.139)<br>P = 0.389	0.986<br>(0.882–1.104)<br>P = 0.811	1.578<br>(1.012–2.461)<br>P = 0.044	1.000<br>(1.000–1.000)<br>P = 0.003	0.997<br>(0.989–1.004)<br>P = 0.357	0.987<br>(0.974–1.000)<br>P = 0.050	0.855<br>(0.415–1.766)<br>P = 0.671	1.006<br>(1.003–1.010)<br>P < 0.001	1.147<br>(0.757 – 1.738)<br>P = 0.516
	Categorical	Q1	1.598<br>(0.543–4.702)<br>P = 0.395	1.094<br>(0.405–2.956)<br>P = 0.859	2.881<br>(0.855–9.702)<br>P = 0.088	3.451<br>(0.808–14.741)<br>P = 0.095	0.898<br>(0.382–2.110)<br>P = 0.804	1.830<br>(0.624–5.369)<br>P = 0.271	0.901<br>(0.357–2.275)<br>P = 0.826	0.863<br>(0.318–2.348)<br>P = 0.774	2.861<br>(0.852–9.616)<br>P = 0.089	1.135<br>(0.467–2.761)<br>P = 0.780
		Med	1.549<br>(0.702–3.420)<br>P = 0.278	1.655<br>(0.743–3.687)<br>P = 0.218	1.040<br>(0.474–2.280)<br>P = 0.923	2.637<br>(1.042–6.675)<br>P = 0.041	1.265<br>(0.577–2.775)<br>P = 0.557	0.648<br>(0.283–1.487)<br>P = 0.306	0.623<br>(0.275–1.412)<br>P = 0.257	0.888<br>(0.397–1.987)<br>P = 0.772	3.161<br>(1.309–7.636)<br>P = 0.011	1.320<br>(0.598–2.913)<br>P = 0.490
		Q3	1.319<br>(0.546–3.183)<br>P = 0.538	1.087<br>(0.430–2.751)<br>P = 0.860	0.767<br>(0.304–1.930)<br>P = 0.573	2.929<br>(1.266–6.776)<br>P = 0.012	0.898<br>(0.382–2.110)<br>P = 0.804	0.764<br>(0.384–2.057)<br>P = 0.594	0.235<br>(0.055–1.000)<br>P = 0.050	0.808<br>(0.334–1.953)<br>P = 0.636	5.319<br>(2.318–12.209)<br>P = 0.001	1.714<br>(0.751–3.913)<br>P = 0.214
Multifocal Recurrence	Non-categorical		1.099<br>(0.793–1.525)<br>P = 0.570	0.993<br>(0.896–1.101)<br>P = 0.899	0.973<br>(0.879–1.077)<br>P = 0.599	1.359<br>(0.938–1.968)<br>P = 0.105	1.000<br>(1.000–1.000)<br>P = 0.005	1.000<br>(0.995–1.004)<br>P = 0.960	0.998<br>(0.992–1.004)<br>P = 0.513	0.728<br>(0.386–1.371)<br>P = 0.325	1.004<br>(1.000–1.008)<br>P = 0.080	1.124<br>(0.776–1.626)<br>P = 0.536
	Categorical	Q1	0.977<br>(0.437–2.185)<br>P = 0.955	0.762<br>(0.350–1.656)<br>P = 0.762	1.153<br>(0.512–2.598)<br>P = 0.732	2.237<br>(0.769 – 6.508)<br>P = 0.139	1.524<br>(0.658–3.530)<br>P = 0.325	2.609<br>(0.793–5.394)<br>P = .137	0.773<br>(0.356–1.680)<br>P = 0.516	0.798<br>(0.336–1.893)<br>P = 0.609	1.594<br>(0.656–3.876)<br>P = 0.752	1.008<br>(0.465–2.183)<br>P = 0.984
		Med	1.568<br>(0.779–3.158)<br>P = 0.482	1.805<br>(0.881–3.698)<br>P = 0.107	0.827<br>(0.427–1.660)<br>P = 0.592	1.248<br>(0.614–2.536)<br>P = 0.540	1.976<br>(0.965–4.047)<br>P = 0.063	0.954<br>(0.470–1.938)<br>P = 0.897	0.887<br>(0.438–1.796)<br>P = 0.887	0.757<br>(0.378–1.519)<br>P = 0.434	1.380<br>(0.681–2.795)<br>P = 0.371	1.010<br>(0.504–2.022)<br>P = 0.978
		Q3	0.991<br>(0.428–2.295)<br>P = 0.984	0.991<br>(0.434–2.329)<br>P = 0.991	0.686<br>(0.296–1.590)<br>P = 0.686	1.925<br>(0.928–3.992)<br>P = 0.079	1.524<br>(0.658–3.530)<br>P = 0.325	1.090<br>(0.483–2.461)<br>P = 0.835	0.745<br>(0.322–1.723)<br>P = 0.491	0.899<br>(0.425–1.899)<br>P = 0.780	1.986<br>(0.909–4.340)<br>P = 0.085	1.483<br>(0.701–3.137)<br>P = 0.302
Survival	Non-categorical		1.731<br>(1.401–2.139)<br>P = 0.001	1.129<br>(1.061–1.202)<br>P <0.001	0.945<br>(0.813–1.098)<br>P = 0.45	1.094<br>(0.714–1.676)<br>P = 0.681	1.000<br>(1.000–1.000)<br>P = 0.001	1.003<br>(0.999–1.007)<br>P = 0.214	0.997<br>(0.988–1.005)<br>P = 0.475	0.643<br>(0.294–1.458)<br>P = 0.291	1.006<br>(1.003–1.008)<br>P = 0.001	1.676<br>(1.181–2.379)<br>P = 0.009
	Categorical	Q1	1.624<br>(0.471–5.599)<br>P = 0.442	1.077<br>(0.356–3.262)<br>P = 0.895	1.420<br>(0.486–4.303)<br>P = 0.536	1.614<br>(0.463–5.621)<br>P = 0.452	3.311<br>(0.763–14.368)<br>P = 0.110	5.585<br>(0.743–41.999)<br>P = 0.095	1.188<br>(0.393–3.593)<br>P = 0.760	0.447<br>(0.174–1.146)<br>P = 0.094	1.712<br>(0.498–5.885)<br>P = 0.394	6.422<br>(0.857–48.138)<br>P = 0.070
		Med	2.524<br>(0.959–6.645)<br>P = 0.061	2.221<br>(0.844–5.845)<br>P = 0.106	1.854<br>(0.703–4.892)<br>P = 0.212	0.985<br>(0.399–2.430)<br>P = 0.973	3.119<br>(1.123–8.666)<br>P = 0.029	1.368<br>(0.547–3.423)<br>P = 0.503	0.721<br>(0.288–1.807)<br>P = 0.486	0.654<br>(0.262–1.632)<br>P = 0.363	2.330<br>(0.885–6.139)<br>P = 0.087	2.479<br>(0.892–6.887)<br>P = 0.082
		Q3	3.290<br>(1.334–8.110)<br>P = 0.010	3.282<br>(1.331–8.094)<br>P = 0.010	0.651<br>(0.215–1.970)<br>P = 0.448	2.087<br>(0.883–5.234)<br>P = 0.117	3.311<br>(0.763–14.368)<br>P = 0.110	1.687<br>(0.654–4.352)<br>P = 0.279	0.511<br>(0.148–1.759)<br>P = 0.287	0.979<br>(0.371–2.579)<br>P = 0.965	5.808<br>(2.325–14.509)<br>P = 0.001	2.662<br>(1.080–6.564)<br>P = 0.033

* Hazard ratio (95%CI)
